# Hepatocyte-Specific *Nrf2* Deficiency Alters Cholesterol Metabolism, Leading to Mitigated Atherosclerosis in *ApoE*-Knockout Mice

**DOI:** 10.3390/antiox15091183

**Published:** 2026-09-17

**Authors:** Junying Jiao, Ning Xu, Zhixuan Hong, Lei Chang, Derong Huang, Yang Yu, Zhendi Wang, Yibai Li, Jiaxin Yu, Juntao Guo, You Wang, Lirun Kuang, Yong Wang, Bei Yang, Rui Zhao, Yongyong Hou, Huihui Wang, Qiang Zhang, Ping Xu, Yuanyuan Xu, Jingbo Pi, Jingqi Fu

**Affiliations:** 1 Key Laboratory of Environmental Stress and Chronic Disease Control & Prevention, China Medical University, Ministry of Education, No. 77 Puhe Road, Shenyang North New Area, Shenyang 110122, China; junyingjiaocmu@163.com (J.J.);; 2 Key Laboratory of Liaoning Province on Toxic and Biological Effects of Arsenic, China Medical University, No. 77 Puhe Road, Shenyang North New Area, Shenyang 110122, China; 3 School of Public Health, China Medical University, No. 77 Puhe Road, Shenyang North New Area, Shenyang 110122, China; 4 State Key Laboratory of Proteomics, Beijing Proteome Research Center, National Center for Protein Sciences (Beijing), Research Unit of Proteomics & Research and Development of New Drug of Chinese Academy of Medical Sciences, Beijing Institute of Lifeomics, Beijing 102206, China; 5 Department of General Surgery, The Fourth Affiliated Hospital of China Medical University, Shenyang 110032, China; 6 School of Basic Medical Sciences, China Medical University, No. 77 Puhe Road, Shenyang North New Area, Shenyang 110122, China; 7 School of Forensic Medicine, China Medical University, No. 77 Puhe Road, Shenyang North New Area, Shenyang 110122, China; 8 Gangarosa Department of Environmental Health, Rollins School of Public Health, Emory University, Atlanta, GA 30322, USA

**Keywords:** hepatocyte, NRF2, cholesterol metabolism, atherosclerosis

## Abstract

Hypercholesterolemia and hyperlipidemia are major contributors to the pathogenesis and progression of atherosclerosis and cardiovascular diseases (CVDs). Nuclear factor erythroid-derived 2-like 2 (NRF2) exerts antioxidant and anti-inflammatory effects while regulating glucose and lipid metabolism. However, the precise role and molecular mechanisms of NRF2 in hepatic cholesterol metabolism remain incompletely understood. Approach and Results: *ApoE*-knockout (*ApoE*-KO) mice were crossed with hepatocyte-specific *Nrf2*-knockout (*Nrf2*(H)-KO) mice to explore how NRF2 modulated cholesterol metabolism and the progression of atherosclerosis in vivo. Atherosclerotic lesion areas in *Nrf2*(H)-KO;*ApoE*-KO mice were significantly reduced compared with control counterparts (*Nrf2*-LoxP;*ApoE*-KO). Total cholesterol and LDL-cholesterol levels in plasma of *Nrf2*(H)-KO;*ApoE*-KO mice were significantly decreased, consistent with atherosclerotic phenotypes. Furthermore, hepatic triglyceride levels and cholesterol contents were increased in *Nrf2*(H)-KO;*ApoE*-KO mice. The hepatic transcriptomic analysis highlighted lipid metabolism pathway alterations in *Nrf2*(H)-KO;*ApoE*-KO mice, and carboxylesterase 1 (*Ces1s*) and lipocalin gene family member major urinary proteins (*Mups*) clusters markedly reduced expression in *Nrf2*(H)-KO;*ApoE*-KO livers. Liver proteomics analysis corroborated the transcriptomic findings. The current findings suggest that NRF2-modulated CES1s and MUPs may regulate cholesterol metabolism in the *ApoE*-KO model. Conclusions: Hepatocyte-specific *Nrf2* deficiency significantly reduced plasma cholesterol levels, thereby attenuating atherosclerotic plaque formation and progression in the *ApoE*-KO background. This study established NRF2 in hepatocytes as a potential therapeutic target for controlling hypercholesterolemia and preventing atherosclerosis development.

## 1. Introduction

Cardiovascular disease (CVD) represents a major global health burden, ranking among the most prevalent chronic non-communicable disorders threatening human health worldwide. In China, CVD accounts for over 40% of mortality among both urban and rural populations [1]. Convergent evidence from epidemiological, genetic, and clinical intervention studies has established low-density lipoprotein cholesterol (LDL-C) as a causal risk factor for atherosclerotic cardiovascular disease (ASCVD) [2]. Halting the rising trend in population-wide serum cholesterol levels remains a critical public health priority for global ASCVD prevention.

The pathogenesis of atherosclerosis involves a series of pathological processes, and pro-inflammatory cytokines and cardiovascular risk factors trigger a cascade of atherogenic events, including subendothelial lipoprotein retention and modification, monocyte recruitment and differentiation, and macrophage-derived foam cell generation [3,4]. Notably, circulating lipoprotein-cholesterol concentrations constitute a modifiable determinant of atherosclerotic progression. Hepatocytes play a key role in regulating plasma lipoprotein metabolism by secreting very low-density lipoprotein (VLDL) and taking up high-density lipoprotein (HDL), low-density lipoprotein (LDL), and chylomicron remnants [5]. Dysregulation of multiple steps in hepatic cholesterol homeostasis—including increased cholesterol biosynthesis, impaired cholesterol efflux and uptake, and enhanced cholesterol esterification—contributes to elevated free cholesterol levels in hepatocytes, which in turn drives the progression of metabolic dysfunction-associated fatty liver disease (MAFLD) and atherosclerosis [6,7].

Nuclear factor erythroid-derived 2-like 2 (NRF2) has emerged as a pivotal regulator in atherosclerosis pathogenesis. Beyond its canonical roles in redox homeostasis and xenobiotic detoxification, NRF2 exerts multifaceted regulation throughout atherosclerosis progression. The NRF2-mediated adaptive antioxidant response is the most important and most studied regulatory mechanism among various cellular defense mechanisms against oxidative stress [8,9]. NRF2 is subjected to ubiquitin-proteasomal degradation via binding to two specific motifs of its negative regulator, KEAP1. Upon exposure to electrophiles or oxidants, NRF2 ubiquitination is blocked by conformational changes in KEAP1, leading to NRF2 accumulation and subsequent transcription of its target antioxidant genes [10,11,12].

Previous studies have shown that NRF2 orchestrates multifaceted regulation of atherosclerosis, including but not limited to lipid metabolism, redox signaling regulation, inflammatory factor release, and reverse cholesterol transport [13,14,15]. Interestingly, NRF2 exhibits context-dependent dual roles in atherosclerosis pathogenesis. NRF2 has been shown to be protective against cardiovascular disease, but it can promote the occurrence and development of atherosclerosis. In three atherosclerosis mouse models (*ApoE^−/^^−^*, *LDLR^−/^^−^*, and *LDLR^−/^^−^*; *ApoB*^100/100^), systemic *Nrf2*-knockout reduced the degree of atherosclerosis [14,16,17]. Systemic *Nrf2* deficiency may have reduced the size of atherosclerotic lesions through systemic effects on lipoprotein profiles. Paradoxically, in aged *Nrf2^−/^^−^LDLR^−/^^−^ApoB*^100/100^ mice, systemic *Nrf2* deficiency leads to enhanced atherosclerotic plaque instability through increased plaque inflammation and oxidative stress, which result in myocardial infarction and sudden death [17]. In contrast, endothelial-specific NRF2 can play an anti-inflammatory role by activating the downstream gene *Ho-1* to reduce the expression of VCAM-1 and MCP-1 [18]. NRF2 also prevents foam cell and atherosclerotic plaque formation by promoting downstream gene expression and activating antioxidant enzymes, such as SOD and GSH-Px, to remove reactive oxygen species (ROS) and lipid peroxidation (LPO) products [19]. In *ApoE^−/^^−^* mice fed a high-cholesterol diet (HCD), knockdown of *Nrf2*, specifically in the endothelium, accelerated plaque formation, accompanied by elevated expression of VCAM-1 and 4-HNE in the vascular endothelium [13,18,20,21]. Macrophage-specific NRF2 plays an important role in lipid uptake and efferocytosis, thereby promoting the occurrence and development of atherosclerosis [14,22]. Meanwhile, in *LDLR^−/^^−^* mice, transplantation of *Nrf2*-specific knockout bone marrow-derived cells increased atherosclerosis [23]. These contradictory results suggest that the impact of NRF2 in atherosclerosis is likely dependent on cell type and genetic background.

Despite these findings, the specific contribution of hepatocyte NRF2 to atherosclerosis remains largely unexplored. Given the liver’s central role in regulating systemic lipid homeostasis, hepatic NRF2 may influence atherogenesis by modulating key lipid-transporting pathways. For instance, carboxylesterases (CESs) have been shown to hydrolyze endogenous esters and thioesters, playing important physiological functions in lipid metabolism and energy homeostasis. Significant decreases in multiple CES isoforms can impair VLDL assembly and secretion, potentially leading to the attenuation of atherosclerosis [24]. Similarly, major urinary proteins (MUPs) are members of the lipid-transporting protein superfamily, which are synthesized predominantly in the liver and secreted into the bloodstream. It has been reported that a significant reduction in MUP isoforms affects hepatic lipid transport into the circulation, thereby influencing the development of atherosclerosis [25]. Building upon these insights, we hypothesized that hepatocyte-specific NRF2 contributes to atherosclerosis progression by modulating cholesterol homeostasis.

## 2. Methods and Materials

### 2.1. Animals and Experimental Design

In this work, we employed a hepatocyte-specific *Nrf2*-knockout mouse model, which was utilized in our group previously [26,27,28]. *Nrf2*(H)-KO mice on a C57BL/6 background were generated by breeding *Nrf2*^LoxP/LoxP^ mice with *Albumin*-Cre mice (J003574, Biomedical Research Institute of Nanjing University, Nanjing, China) [26,28]. Apolipoprotein E-deficient (*ApoE*-KO) mice (Cat. No. NM-KO-00033) were purchased from Shanghai Model Organisms Center, Inc (Shanghai, China). All breeders and littermates were housed at the animal facility of China Medical University under specific pathogen-free (SPF) conditions. All animal experiments were approved by the Institutional Animal Care and Use Committee of China Medical University (CMU20231089). The mice were housed in temperature-controlled cages with a 12/12 h light/dark cycle and had free access to water and a standard chow diet unless otherwise specified.

In this study, 24-week-old male mice, 60-week-old male mice, and 24-week-old female mice were utilized (Figure S12). For the experimental design, mice were randomly divided into four groups: (1) *Nrf2*-LoxP;*ApoE*-WT, (2) *Nrf2*(H)-KO;*ApoE*-WT, (3) *Nrf2*-LoxP;*ApoE*-KO, and (4) *Nrf2*(H)-KO;*ApoE*-KO. The body weight of the mice was monitored regularly. For the determination of fasting blood glucose, mice were fasted overnight with free access to water. Blood samples were collected from the tail vein, and blood glucose levels were immediately measured using a standard glucometer. At the designated end points, the animals were sacrificed, and relevant organs, including the liver, spleen, kidney, subcutaneous adipose tissues, epididymal white adipose tissues and brown adipose tissues, were rapidly excised, carefully rinsed in ice-cold PBS, blotted dry, and precisely weighed to calculate the organ-to-body weight ratios.

### 2.2. Analysis of Atherosclerosis

Mice were euthanized using CO_2_, and then the entire aorta, including the aortic arch, thoracic, and abdominal regions, was stripped and fixed in 4% paraformaldehyde for more than 48 h. The aorta was incised longitudinally to expose the atherosclerotic plaque and fixed on a black background plate. Aortic tissue was washed three times with PBS, then rinsed twice with 60% isopropanol, and stained with Oil Red O (G1262, Solarbio, Beijing, China) for 30 min. Then, the aorta was rinsed with 60% isopropanol (40064360, Sinopharm Chemical Reagent Co., Ltd., Shanghai, China) until the non-specific staining was washed out completely [21,22]. Images were captured for further analysis, and plaque area quantification was performed using ImageJ software (version 1.54p). Percentage plaque area was calculated as the ratio of plaque area to total vessel area.

### 2.3. Measurement of Plasma and Hepatic Cholesterol and Triglyceride Levels

Fresh blood was collected via ocular puncture following a 16 h fast in mice. Heparin-treated fresh blood samples were centrifuged at 5000× *g* for 5 min at 4 °C to collect plasma samples. The liver samples were homogenized in chloroform (10006818, Sinopharm Chemical Reagent Co., Ltd.): methanol (40064260, Sinopharm Chemical Reagent Co., Ltd.) = 2:1, and then centrifuged at 4200× *g* for 5 min at 4 °C. After aspirating the lower layer of liquid, it was used for subsequent measurements. The levels of total triglyceride (TG) (Cat# A110-1-1, Nanjing Jiancheng Bioengineering Institute, NJJCBIO), total cholesterol (T-CHO) (Cat# A111-1-1, NJJCBIO), LDL-C (Cat# A113-1-1, NJJCBIO), HDL-C (Cat# A112-1-1, NJJCBIO), nonesterified free fatty acids (NEFAs) (Cat# A042-1-1, NJJCBIO), glycerol (Cat# F005-1-1, NJJCBIO) and other relevant indicators in plasma samples and hepatic lipid extracts were assessed using specific kits from NJJCBIO (Nanjing, China) according to the manufacturer’s instructions.

### 2.4. Western Blotting

Liver tissues were lysed according to a standard protocol, and protein determination and Western blotting were completed as previously described [29]. Antibodies for NRF2 (sc-13032; 1:800; Santa Cruz Biotechnology, Dallas, TX, USA), CES1 (ab45957; 1:1000; Abcam, Waltham, MA, USA), MUPs (sc-166429; 1:1000; Santa Cruz Biotechnology), APOB (ab31992; 1:1000; Abcam), α-TUBULIN (M20005S; 1:2000; Abmart, Shanghai, China) and β-ACTIN (sc-47778; 1:1000; Santa Cruz Biotechnology) were used, with the last two as loading controls.

### 2.5. Histological Examination

Liver samples were dissected and fixed in 4% paraformaldehyde for at least 48 h. Then, tissues were dehydrated through a graded ethanol series and embedded in paraffin. Sections at 3 μm thickness were obtained via the HistoCore AUTOCUT R-Automated Rotary Microtome (Leica, Nussloch, Germany) followed by H&E staining. An 8 μm thick frozen section was used for Oil Red O staining. For IHC staining, the primary antibody against MUPs (sc-166429; 1:1000, Santa Cruz) was used and followed by DAB staining (ZLI-9019, Zhongshan Golden Bridge BioTech, Co., Ltd., Beijing, China). Pathological alterations were analyzed using an optical microscope (200×, DMi8, Leica).

### 2.6. Hepatic VLDL-TG Secretion Assay

To assess the hepatic very low-density lipoprotein secretion rate, the mice were fasted for 4 h and subsequently injected intraperitoneally with 1 g/kg body weight of Poloxamer-407 (P434419, Aladdin, Shanghai, China). Blood samples were harvested from the tail vein before administration (0 h) and at 1, 2, 3 and 4 h after the injection of Poloxamer-407. TG levels were determined at the indicated time points [30].

### 2.7. Plasma Untargeted Metabolomics Analysis

The plasma samples were thawed at 4 °C, and 100 μL aliquots were mixed with 400 μL of cold methanol/acetonitrile (34851, Sigma, Shanghai, China) (1:1, *v*/*v*) to precipitate proteins. The mixture was centrifuged for 20 min (14,000× *g*, 4 °C). The supernatant was dried in a vacuum centrifuge. For LC-MS analysis, the samples were redissolved in 100 μL of acetonitrile/water (1:1, *v*/*v*) solvent and centrifuged at 14,000× *g* at 4 °C for 15 min, and then the supernatant was injected. Analysis was performed using a UHPLC (Vanquish UHPLC, Thermo, Shanghai, China) coupled to an Orbitrap in Shanghai Applied Protein Technology Co., Ltd. (Shanghai, China). The raw data were converted to the “.mzXML” format by ProteoWizard (version 3.0.21229), and then XCMS software (version 3.12.0) was used for peak alignment, retention time correction, and peak area extraction. The data extracted by XCMS were first subjected to metabolite structure identification and data pre-processing (null filtering: removing ion peaks with missing values > 50%; null filling: k-nearest neighbors (KNN) imputation; data filtering: removing features with RSD > 50%). Experimental data were then evaluated by data quality (6 quality controls) and analyzed.

### 2.8. Quantitative RT-PCR Analysis and Liver RNA Sequencing and Analysis

RNA was extracted from tissues or cellular samples using RNAiso Plus (Takara, #9109). Total RNA was reverse transcribed into cDNA using PrimeScript RT Master Mix (Takara, RR036A). Real-time quantitative PCR reactions were performed using the SYBR Premix EX Taq Kit (Takara, Dalian, China) and the QuantStudio 6 Flex real-time PCR system (Applied Biosystems, Waltham, MA, USA). All mRNA levels were presented relative to *Actin* of *Nrf2*-LoxP livers.

RNA-seq was carried out by Seqhealth Technology Co., Ltd. (Wuhan, China). Total RNA was extracted from the mouse liver. After RNA quality evaluation and library preparation, the library products were further sequenced with the Illumina NovaSeq 6000 sequencing platform (San Diego, CA, USA). Differentially expressed genes (DEGs) were screened using thresholds of |log2 (fold change)| > 1 and *p* < 0.05.

### 2.9. Liver Proteomics Analysis

Proteomics analysis was carried out by the National Center for Protein Sciences (Beijing, China). The parameters for protein extraction, digestion, iTRAQ labeling, RP-HPLC peptide separation, and LC-MS/MS analysis have been described previously [31]. The criteria for significantly differentially expressed proteins (DEPs) were set at *p* < 0.05 and fold change > 1.5 or < 0.67.

### 2.10. Public ChIP-Seq Dataset Analysis

Published chromatin immunoprecipitation sequencing (ChIP-seq) data for NRF2 and H3K27ac were retrieved from the NCBI BioProject database (accession numbers: PRJNA806849 and PRJNA345270). Targeted analysis was performed on the genomic loci of the *Mups* and *Ces1s* genes [32,33].

### 2.11. Statistical Analysis

All data were expressed as mean ± standard deviation. GraphPad Prism (Version 9.0, Boston, MA, USA) was used to analyze the data. For comparing two independent groups, we applied the Mann–Whitney test, and for comparing multiple groups, we used the Kruskal–Wallis test, followed by the Dunn multiple comparison test. Pearson correlation analysis was performed to evaluate the linear correlation between two continuous variables. *p* < 0.05 was considered statistically significant.

## 3. Results

### 3.1. Nrf2 Deficiency in Hepatocytes Reduced Atherosclerotic Lesion Progression

**Figure 1:**
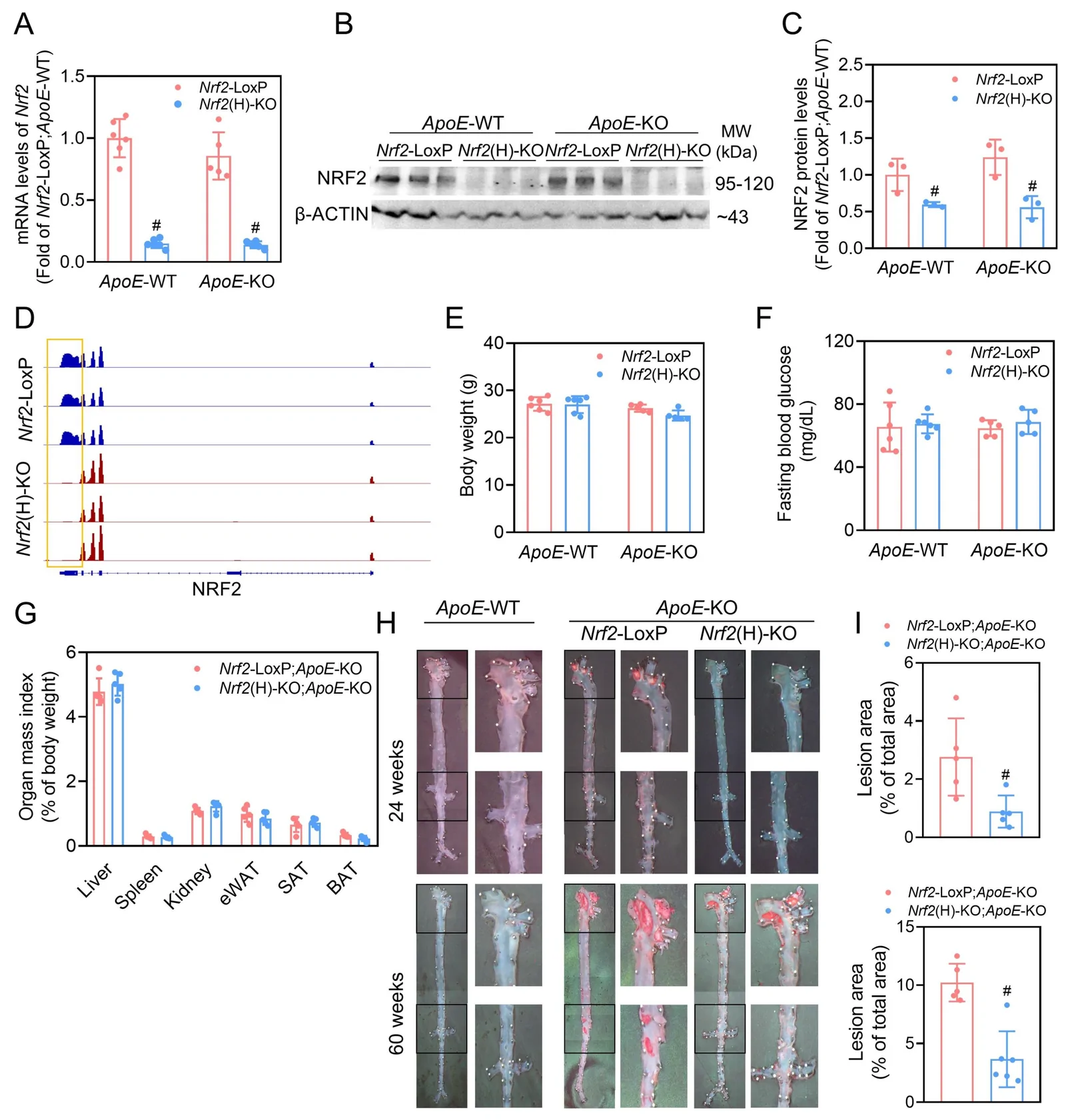
*Nrf2* deficiency in hepatocytes attenuated atherosclerotic progression in male *ApoE*-KO mice. (**A**) Hepatic *Nrf2* mRNA levels. (**B**) Representative image of immunoblots of NRF2 using the liver tissues. (**C**) Relative quantitative protein levels of NRF2. (**D**) Genome coverage plot of the *Nrf2* locus. (**E**) Body weight, (**F**) fasting blood glucose, (**G**) organ mass index in 24-week-old *Nrf2*-LoxP and *Nrf2*(H)-KO mice in the *ApoE*-KO background. (**H**) Oil-red O staining of the aorta in male mice of different ages (24 weeks old and 60 weeks old). (**I**) Quantification of Oil-red O staining of aorta. Values are mean ± SD. *n* = 5–6, ^#^ *p* < 0.05 vs. *Nrf2*-LoxP group of the same *ApoE* genotype.

To determine the definitive role of hepatocyte NRF2 in the pathogenesis of atherosclerosis, we generated *Nrf2*(H)-KO and littermate control *Nrf2*-LoxP mice in the *ApoE*-KO background. To confirm the knockout efficiency of *Nrf2*(H)-KO mice, mRNA levels and immunoblotting determinations targeting NRF2 were performed using tissue lysates from the liver. As shown in Figure 1A–C, mRNA and protein levels of NRF2 were markedly diminished in the livers of male *Nrf2*(H)-KO mice relative to littermate male *Nrf2*-LoxP controls. We generated the genome coverage plot of the *Nrf2* locus using our RNA-seq data. The plot clearly demonstrates the specific deletion of *Nrf2* exon 5 in *Nrf2*(H)-KO mice, providing direct genomic evidence for the successful generation of our mouse model (Figure 1D). To determine the potential effects of *Nrf2* ablation in hepatocytes on systemic metabolism, body weight and fasting blood glucose levels were assessed longitudinally in all groups regularly for 24-week-old male mice. As shown in Figure 1E,F, there was no significant difference in the basal information of mice among the four groups. To further determine the effect of *Nrf2* deletion on body composition, organ mass index was determined in male *Nrf2*(H)-KO mice and their male *Nrf2*-LoxP controls. Using tibial length to normalize relative organ weights, as shown in Figure 1G, there were no significant differences in organ indices between groups.

*ApoE*-KO mice represent a well-established model for studying atherosclerosis. We compared the extent of atherosclerotic lesion progression throughout the arteries, focusing on the aortic arch and the vessel branches. We found significantly less atherosclerotic progression in male 24-week-old and 60-week-old *Nrf2*(H)-KO;*ApoE*-KO mice, characterized by a smaller area of plaque cores, when compared with littermate *Nrf2*-LoxP;*ApoE*-KO controls (Figure 1H,I).

### 3.2. Nrf2 Deficiency in Hepatocytes Reduced Plasma LDL-C Levels

**Figure 2:**
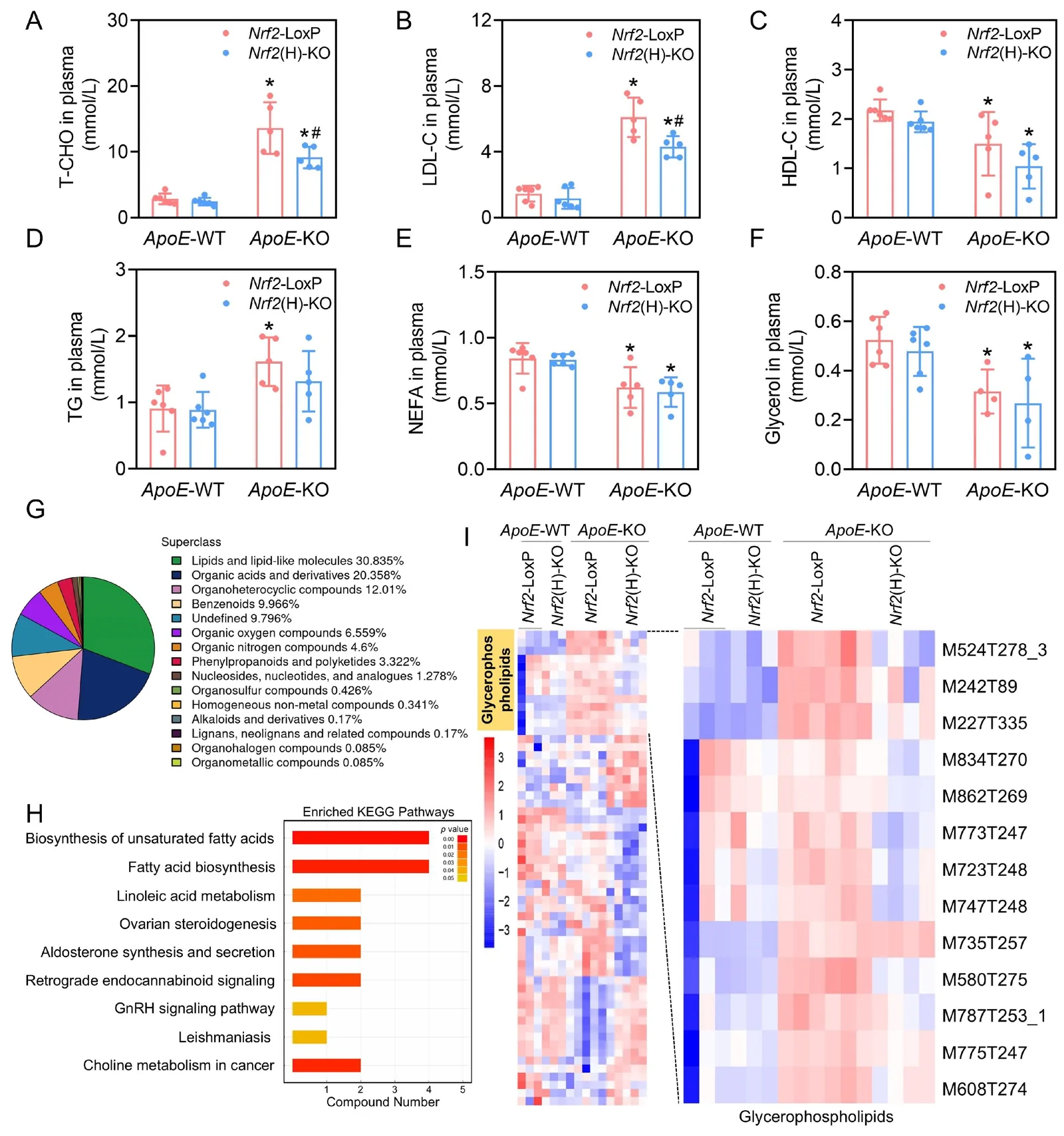
Hepatocyte-specific *Nrf2* deficiency alters plasma lipid profiles. (**A**–**F**) Total cholesterol (T-CHO), low-density lipoprotein cholesterol (LDL-C), high-density lipoprotein cholesterol (HDL-C), total triglyceride (TG), glycerol, nonesterified fatty acids (NEFA) in plasma of 24-week-old male *ApoE*-KO mice. *n* = 5–6, * *p* < 0.05 vs. *ApoE*-WT group, ^#^ *p* < 0.05 vs. *Nrf2*-LoxP group. (**G**) Classification and relative proportions of all identified metabolites. (**H**) Enriched KEGG pathways of differential metabolites in *Nrf2*(H)-KO;*ApoE*-KO mice and *Nrf2*-LoxP;*ApoE*-KO mice. (**I**) Heatmap of differential metabolites in *Nrf2*(H)-KO;*ApoE*-KO mice and *Nrf2*-LoxP;*ApoE*-KO mice. All metabolites related to glycerophospholipids are listed therein. (M524T278_3: 1-Stearoyl-sn-glycerol 3-phosphocholine(LPC (18:0)), M242T89: Dimethachlor cga369873, M227T335: Biotin, M834T270: Pi 34:2, M862T269: Pi 36:2, M773T247: (2-aminoethoxy) [2-[docosa-4.7.10.13.16.19-hexaenoyloxy]-3-[octadeca-1.9-dien-1-yloxy]propoxy] phosphinic acid, M723T248: (2-aminoethoxy) [3-[hexadec-1-en-1-yloxy]-2-[icosa-5.8.11.14-tetraenoyloxy] propoxy]phosphinic acid, M747T248: (2-aminoethoxy) [2-[docosa-4.7.10.13.16.19-hexaenoyloxy]-3-[hexadec-1-en-1-yloxy]propoxy]phosphinic acid, M735T257: 1,2-dihexadecanoyl-sn-glycero-3-phosphocholine, M580T275: 1-behenoyl-2-hydroxy-sn-glycero-3-phosphocholine, M787T253_1: 1-stearoyl-2-linoleoyl-sn-glycero-3-phosphocholine, M775T247: 1-(1z-octadecenyl)-2-(4z,7z,10z,13z,16z,19z-docosahexaenoyl)-sn-glycero-3-phosphoethanolamine, M608T274: 1-lignoceroyl-2-hydroxy-sn-glycero-3-phosphocholine).

Plasma lipids were monitored dynamically in male mice (Figure S1A–D), which showed that significant differences in cholesterol levels began to appear in *Nrf2*(H)-KO;*ApoE*-KO mice compared with control mice at around 12 weeks, especially LDL-C, and the difference increased significantly with age. We observed the emergence of atherosclerosis at 24 weeks of age. As shown in Figure 2A–D, we found that *Nrf2*(H)-KO;*ApoE*-KO mice had significantly lower levels of total cholesterol in plasma than the control group. In *Nrf2*(H)-KO;*ApoE*-KO mice, plasma LDL-C levels were significantly lower, whereas HDL-C levels remained largely unchanged. We also determined the plasma levels of other lipids in mice, which included triglycerides, glycerol, and free fatty acid levels, and there were no significant differences between the groups. The severity of atherosclerosis echoed the results of less T-CHO in *Nrf2*(H)-KO;*ApoE*-KO mice. In contrast, the difference in 24-week-old female mice was not as significant as in males (Figure S2A–D). In 60-week-old male mice, the differences in lipids between *Nrf2*(H)-KO;*ApoE*-KO mice and their control mice were not significant, except T-CHO (Figure S3A–D). Therefore, we investigated the detailed mechanism of hepatocyte NRF2 in atherosclerosis using 24-week-old male mice in subsequent studies.

In order to investigate the differences in metabolites in the plasma, we performed a high-resolution non-targeted metabolomics analysis of 24-week-old male mice plasma. All metabolites identified in this project (those identified by combining positive and negative ions) were classified and statistically analyzed based on their chemical taxonomy attribution information, and the proportion of the number of each type of metabolite was calculated. Lipids and lipid-like molecules account for 31% of all differential metabolites, as shown in the Figure 2G. Due to the major difference in the degree of atherosclerosis between *Nrf2*(H)-KO;*ApoE*-KO mice and their control *Nrf2*-LoxP;*ApoE*-KO mice, we subjected the differential metabolites of these two groups to KEGG pathway enrichment analysis. Figure 2H shows that two metabolic pathways (the biosynthesis of fatty acids and the biosynthesis of unsaturated fatty acids) were significantly enriched. Figure 2I indicates the results of the hierarchical clustering heatmap of significant differential metabolite levels in *Nrf2*(H)-KO;*ApoE*-KO mice and their control mice, which showed that plasma levels of glycerophospholipid species metabolites were reduced in *Nrf2*(H)-KO;*ApoE*-KO mice compared with controls.

### 3.3. Hepatocyte Nrf2 Gene Deletion Led to Hepatic Lipid Accumulation

**Figure 3:**
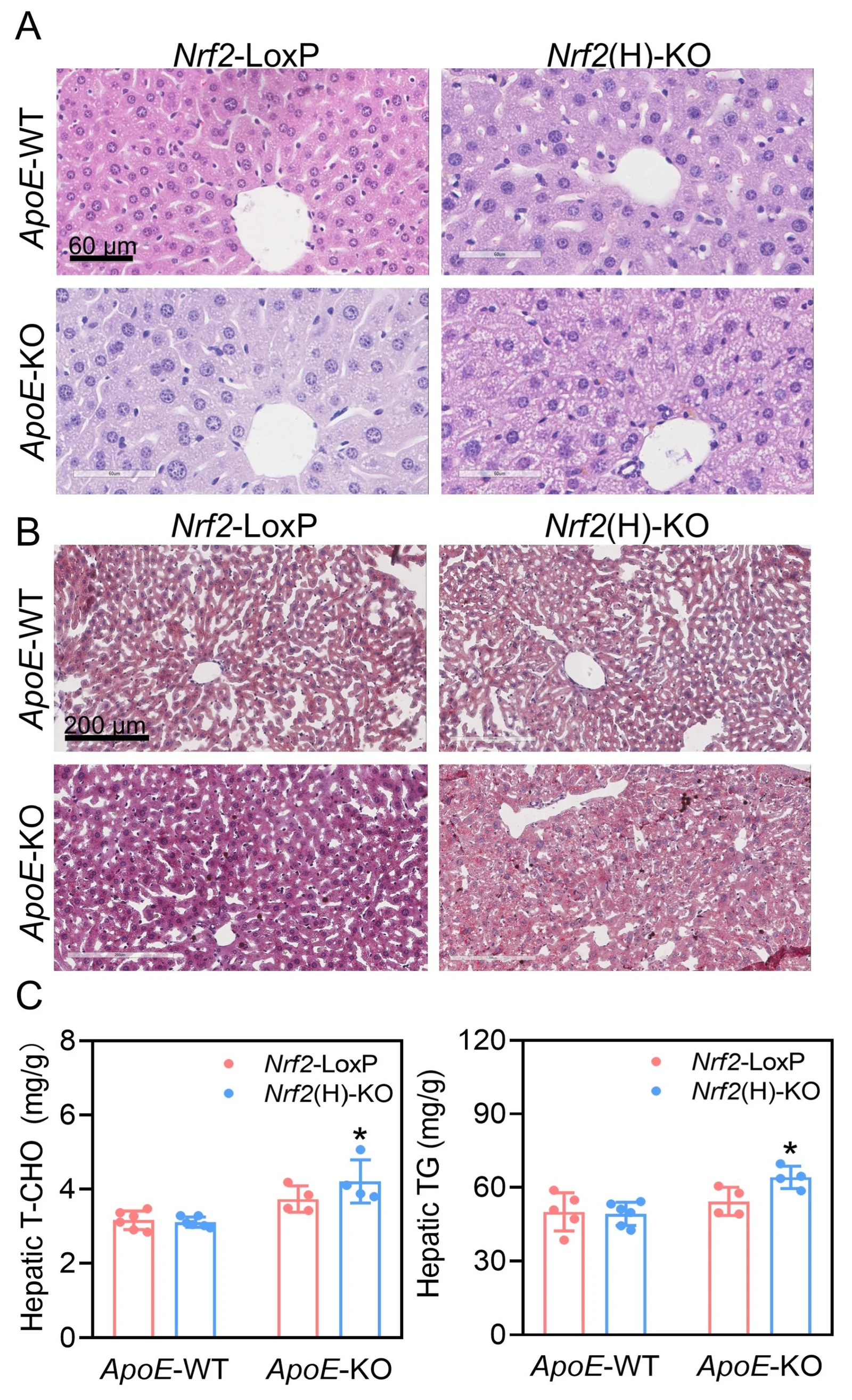
Hepatocyte *Nrf2* deletion led to hepatic lipid accumulation. (**A**) Representative histological images of H&E staining of the livers. Scale bar = 60 μm. (**B**) Representative histological images of Oil-red O staining of the livers. Scale bar = 200 μm. (**C**) Hepatic triglyceride (TG) and total cholesterol (T-CHO) levels. *n* = 4–6, * *p* < 0.05 vs. *ApoE*-WT group of the same *Nrf2* genotype.

Mouse hepatocyte *Nrf2* gene deletion affects atherosclerotic progression as well as plasma lipid content. This result suggests that lipid homeostasis is changed in both the liver and plasma. We assessed this by histologic analysis, and HE staining of livers showed microvesicular steatosis in hepatocytes from male *Nrf2*(H)-KO;*ApoE*-KO mice. The Oil Red O staining of frozen liver sections also revealed an increase in hepatic lipid content in *Nrf2*(H)-KO;*ApoE*-KO mice (Figure 3A,B). To further clarify the hepatic lipid accumulation, we determined the triglyceride and total cholesterol contents in the liver tissues of mice. The results showed that *Nrf2*(H)-KO;*ApoE*-KO mice had elevated intrahepatic triglyceride and total cholesterol accumulation compared with other groups (Figure 3C). These results indicate that although *ApoE*-KO mice develop severe atherosclerosis, the mouse liver does not undergo significant pathological changes. In contrast, in the context of *ApoE*-KO, hepatocyte *Nrf2*-specific deletion leads to microvesicular steatosis in mouse livers.

### 3.4. Hepatocyte Nrf2 Gene Deletion Reduced the Expression of Hepatic Cholesterol Metabolism-Related Genes and Proteins

**Figure 4:**
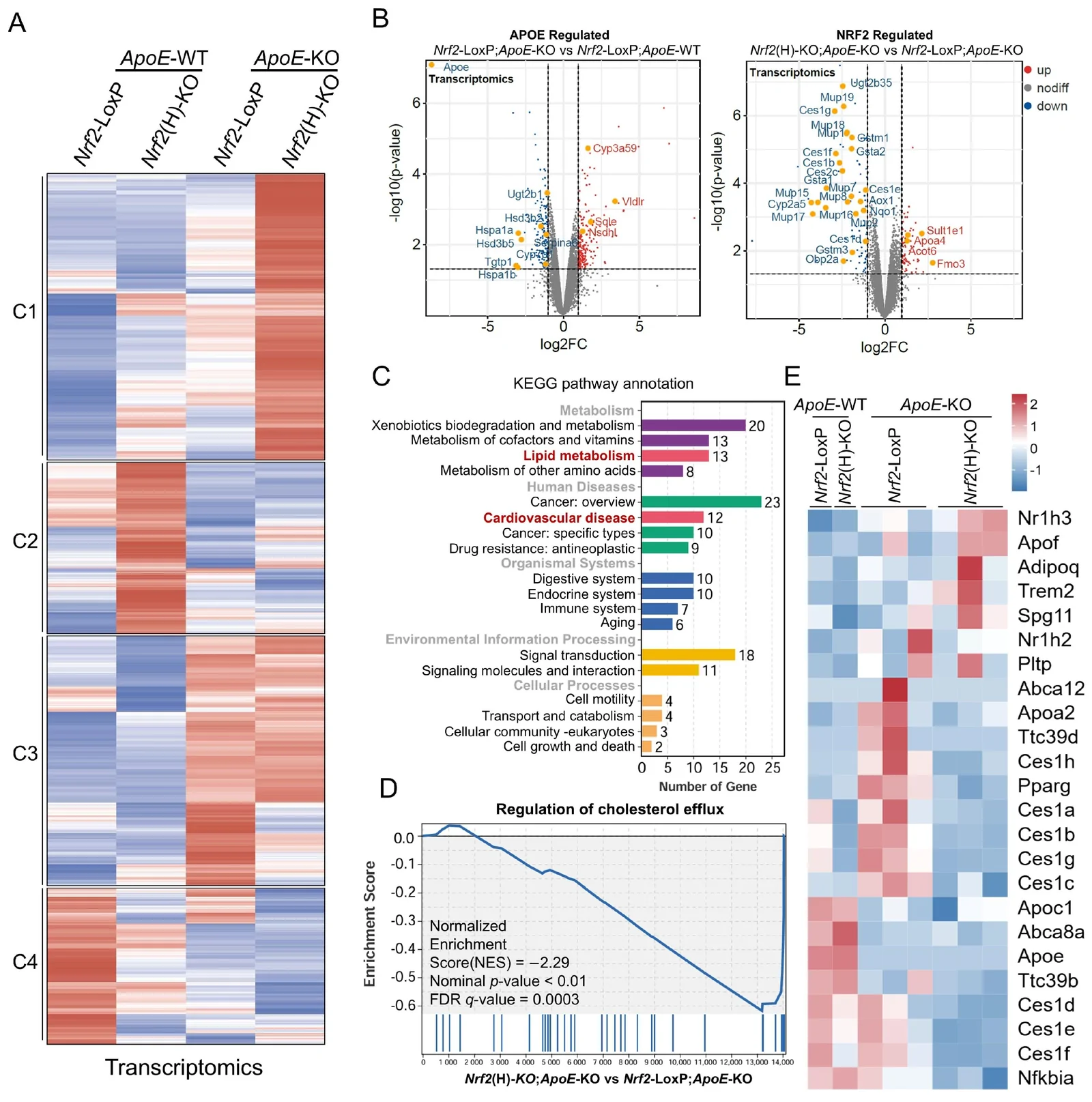
Transcriptomic analyses highlight significant changes in the livers of hepatocyte-specific *Nrf2* deletion in the *ApoE*-KO background. (**A**) The results of a fuzzy c-means clustering analysis combined with a gene ontology enrichment analysis of DEGs are shown. Fuzzy c-means clustering classified all DEGs into four clusters, including regulation of tumor necrosis factor production (C1), fatty acid metabolic process (C2), cellular response to xenobiotic stimulus (C3), and defense response to protozoans and reverse cholesterol transport (C4). (**B**) Genes regulated by APOE (*Nrf2*-LoxP;*ApoE*-KO vs. *Nrf2*-LoxP;*ApoE*-WT) and NRF2 (*Nrf2*(H)-KO;*ApoE*-KO vs. *Nrf2*-LoxP;*ApoE*-KO), respectively, in transcriptomics. (**C**) KEGG enrichment analysis of NRF2-regulated genes. (**D**) GSEA of cholesterol efflux transcriptional network. (**E**) Heatmap of DEGs in cholesterol efflux signaling pathway enrichment maps using GSEA (MSigDB 2026.1). (GOBP_CHOLESTEROL_EFFLUX, https://www.gsea-msigdb.org/gsea/msigdb/mouse/geneset/GOBP_CHOLESTEROL_EFFLUX, 30 January 2026).

To investigate the role of hepatocyte NRF2 in atherosclerosis and hepatic lipid metabolism, we harvested liver tissues from four groups of 24-week-old male mice under fasting conditions. We performed liver transcriptomics analysis combined with proteomic analysis. A total of 29,863 genes were identified through transcriptomic sequencing. We then analyzed differential expression and clustered all differentially expressed genes together using a fuzzy c-means clustering algorithm to identify sets of genes with similar expression patterns. As shown in Figure 4A, we clustered the genes into four categories. Cluster C1 contained 911 genes with higher expression levels in the *Nrf2*(H)-KO;*ApoE*-KO group, mainly associated with pathways such as regulation of tumor necrosis factor production. Cluster C2 contained 545 genes with higher expression levels in the *Nrf2*(H)-KO;*ApoE*-WT group, primarily involved in pathways such as the fatty acid metabolic process. Cluster C3 contained 794 genes with higher expression levels in the *ApoE*-KO group related to cellular response to xenobiotic stimulus pathways. Cluster C4 contained 497 genes with lower expression levels in the *Nrf2*(H)-KO;*ApoE*-KO group, primarily involved in pathways such as defense response to protozoans and reverse cholesterol transport (Figure S4). To compare the changes in liver genes after *ApoE* deletion and *Nrf2* deletion in hepatocytes, we plotted two volcano maps with key upregulated and downregulated genes labeled in the maps. Figure 4B shows the alterations of genes in the liver after *ApoE* deletion, and it can be seen that *ApoE* is significantly downregulated, which confirms that our model is successful. *Nrf2* deletion also significantly reduces its downstream genes, including NAD(P)H quinone oxidoreductase 1 (*Nqo1*); Glutathione S-transferases (*GSTs*), a gene family related to detoxification and xenobiotic metabolism; and UDP-glucuronosyl transferases (*UGTs*). In addition to the above gene families, we also observed significant reductions in genes such as major urinary proteins (*Mups*), a family of lipid transport proteins, and carboxylesterases (*Cess*). We obtained 171 significantly altered differential genes for enrichment analysis comparing *Nrf2*(H)-KO and *Nrf2*-LoxP mice in the *ApoE*-KO background. KEGG enrichment analysis revealed alterations in lipid metabolism and changes in cardiovascular disease, which coincided with our conclusions (Figure 4C). Gene set enrichment analysis (GSEA) further revealed that *Nrf2* deficiency was closely associated with bile acid metabolism, cholesterol metabolism, and regulation of lipid storage (Figure S7A). We further focused on cholesterol metabolism due to the correlation between atherosclerosis and cholesterol metabolism. We found that pathways related to cholesterol metabolism, bile acid metabolism, and cholesterol efflux were significantly downregulated upon *Nrf2* deficiency (Figure 4D). At the same time, we enumerated genes related to cholesterol efflux and showed that most of the genes had reduced expression in *Nrf2*(H)-KO;*ApoE*-KO mice (Figure 4E).

To gain a global perspective on changes in protein abundance, we performed quantitative proteomic analysis on the four experimental cohorts, successfully identifying 4126 protein groups. Pearson correlation analysis demonstrated high concordance among biological replicates. Similar to our transcriptomic findings, Cluster C2 contained 80 proteins with lower expression levels in the *Nrf2*(H)-KO;*ApoE*-KO group, primarily involved in pathway response to stilbenoids (Figure S5). Quantitative proteomics confirmed *ApoE* depletion (Figure S6B), validating knockout efficiency at the translational level. The protein levels of MUPs and CESs are similarly decreased after *Nrf2* deficiency. Consistent with transcriptional findings, proteomic pathway analysis recapitulated lipid metabolic reprogramming and cardiovascular pathogenesis signatures (Figure S6C). Proteomic GSEA delineated pathway divergence, with very long-chain fatty acid catabolism demonstrating activation, contrasting with suppressed bile acid biosynthesis and sterol homeostasis pathways (Figure S7B).

The above analysis shows that the results of transcriptome sequencing and proteome sequencing in the livers of the two groups of mice are in high agreement. It can be seen that cholesterol metabolism-related pathways, including cholesterol efflux-related indexes, are significantly downregulated after *Nrf2* deficiency.

### 3.5. Hepatocyte NRF2 Was Involved in Atherogenesis by Affecting Lipoprotein Secretion

**Figure 5:**
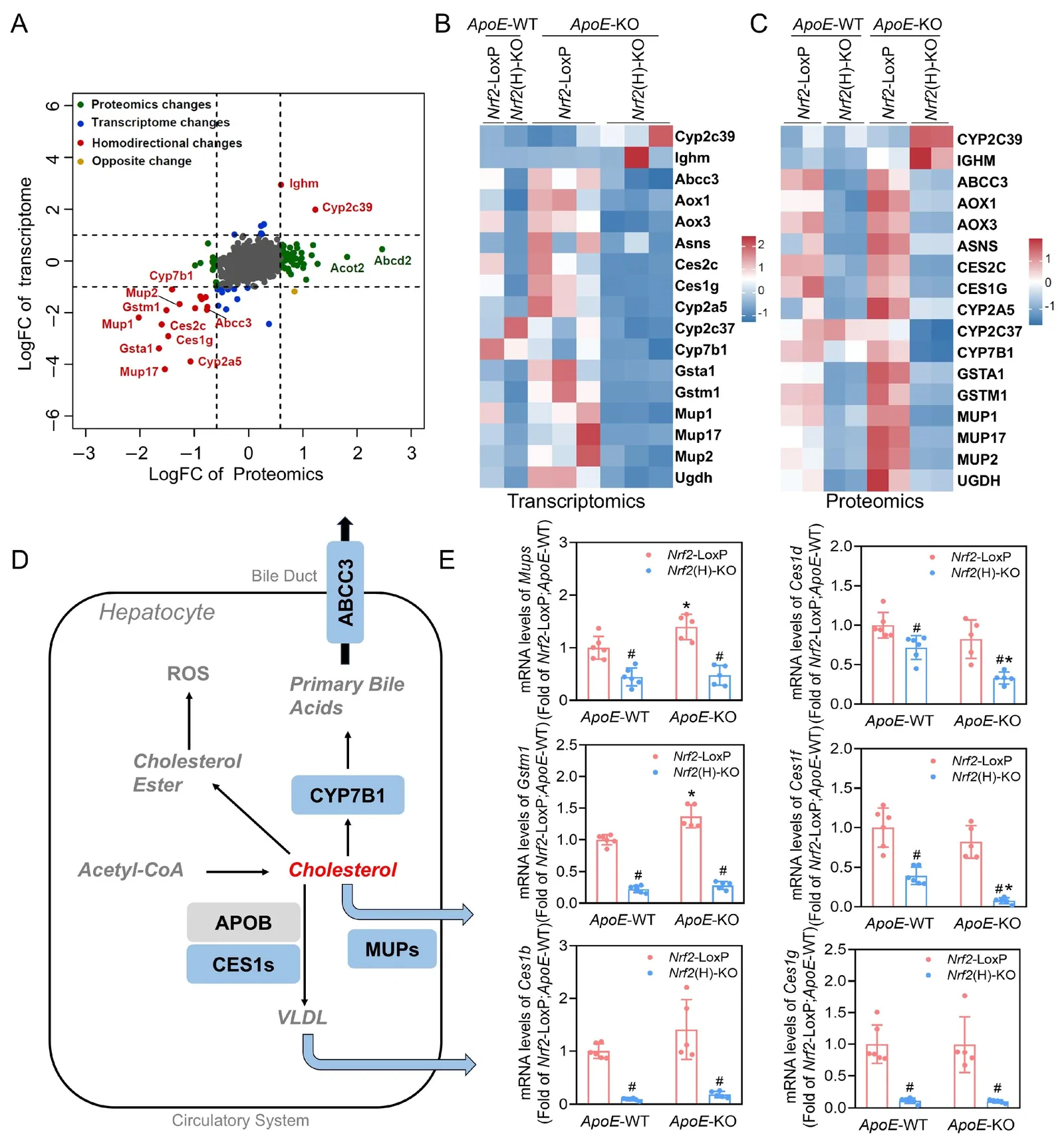
mRNAs and proteins levels were consistently regulated by hepatocyte *Nrf2* gene deletion. (**A**) Both transcriptomics and proteomics analyses were associated and plotted in nine quadrants. (**B**,**C**) Transcriptomics and proteomics isochronous changes in mRNA levels and proteins. (**D**) The process of cholesterol metabolism, in which both genes and proteins are involved, was examined, and blue indicates reduced expression of both genes and proteins. (**E**) The indicated gene expression was confirmed by RT-qPCR. *n* = 5–6, * *p* < 0.05 vs. *ApoE*-WT group of the same *Nrf2* genotype, ^#^ *p* < 0.05 vs. *Nrf2*-LoxP group of the same *ApoE* genotype.

We then jointly analyzed the transcriptomic data and proteomic data and found that 17 genes were significantly changed at both mRNA and protein expression levels in *Nrf2*(H)-KO compared with *Nrf2*-LoxP mice in the *ApoE*-KO background, of which 15 genes were downregulated in both mRNA and protein expression (Figure 5A). We list all 17 of these genes and proteins (Figure 5B,C). Besides the NRF2 downstream-GSTs/UGTs family involved in detoxification metabolic functions, we observed significant downregulation of multiple isoforms within the CESs family in *Nrf2*(H)-KO mice. Furthermore, the MUPs family was similarly affected by hepatocyte *Nrf2* deletion, showing marked reductions across multiple isoforms. In parallel, key regulators of bile acid metabolism, such as CYP7B1 and ABCC3, along with their related transporter genes (*Abcg5/Abcg8*), exhibited reduced expression in *Nrf2*-deficient mice (Figure S8B). The metabolic flow diagram is shown in Figure 5D. The transcriptomics sequencing results were validated by qPCR determination, confirming that *Mups* and *Cess* expression was reduced in *Nrf2*(H)-KO mice (Figure 5E).

**Figure 6:**
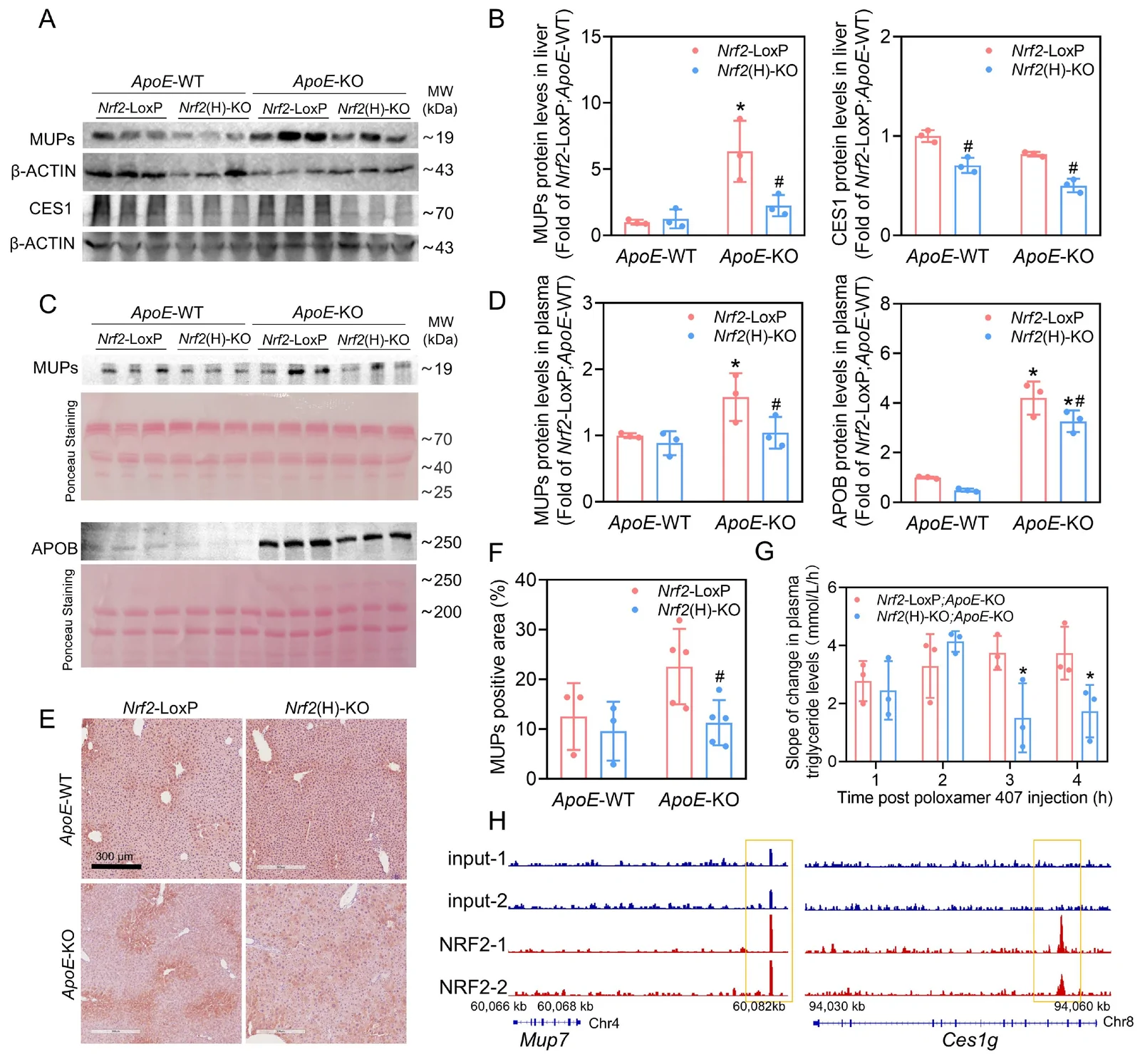
Hepatic lipoprotein secretion was reduced in hepatocyte-specific *Nrf2* deletion in *ApoE*-KO mice. (**A**,**B**) MUP and CES1 protein levels in mouse livers were detected by immunoblots, and the quantitative results are presented, respectively. (**C**,**D**) MUP and APOB protein levels in mouse plasma were detected by immunoblots, and the quantitative results are presented, respectively. (**E**,**F**) MUPs immunohistochemical staining in mouse livers and the quantitative results. (**G**) Hepatic VLDL-TG secretion rate was determined by Poloxamer 407 injection test. (**H**) IGV tracks for *Mup7* and *Ces1g* from ChIP-seq analysis (PRJNA806849). The yellow boxes indicated significant NRF2 binding peaks. *n* = 3–5, * *p* < 0.05 vs. *ApoE*-WT group of the same *Nrf2* genotype, ^#^ *p* < 0.05 vs. *Nrf2*-LoxP group of the same *ApoE* genotype.

We performed immunoblotting on the livers of four groups of 24-week-old male mice, and Western blotting showed that MUPs protein levels were elevated in the *ApoE*-KO background. In contrast, *Nrf2* deficiency significantly reduced the expression levels of MUPs in the liver (Figure 6A,B). Because MUPs, as lipid transport proteins, can be secreted from the liver into the blood, we examined the protein levels of MUPs in plasma, and the results were consistent with those in the liver (Figure 6C,D). We performed immunohistochemical staining in the liver, and we could see that the expression of MUPs was significantly increased in *ApoE*-KO mice and distributed around the central vein. However, after hepatocyte *Nrf2* deficiency, the expression of MUPs decreased, and the distribution became irregular (Figure 6E,F). Similarly, we examined the protein levels of CES1 in the liver, and Western blotting showed that *Nrf2* deficiency significantly reduced the expression levels of CES1 in the liver (Figure 6A). Immunohistochemical results showed the expression of CES1 was significantly increased in *ApoE*-KO mice, and after hepatocyte *Nrf2* deficiency, the expression of CES1s decreased, and the distribution became irregular (Figure S8A).

We also found that APOB protein levels were extremely high in the plasma of *ApoE*-KO mice, which was consistent with the hyperlipidemia of *ApoE*-KO mice. In contrast, APOB expression was reduced in the plasma of *Nrf2*(H)-KO mice (Figure 6C,D). To assess whether NRF2 affects VLDL secretion, we injected poloxamer-407, an inhibitor of lipoprotein lipase and thus of peripheral VLDL-TG hydrolysis, into mice and monitored plasma TG levels. *Nrf2*(H)-KO;*ApoE*-KO mice displayed significantly decreased slope of change in plasma TG levels at 3 h post-injection (Figure 6G). To clarify the difference in LDL uptake, we screened LDL uptake-related genes from the RNA-seq data for analysis, and the corresponding heatmap of key genes (*Ldlr, Apob, Apoe, Lrp1, Pcsk9, Mylip, Nr1h3, Nr1h4)* are presented, demonstrating that hepatocyte-specific *Nrf2* deficiency had no statistically significant effect on LDL uptake (Figure S10B).

To explore the potential regulatory relationship between NRF2 and the *Mups* and *Ces1s* gene families, this study employed chromatin immunoprecipitation sequencing (ChIP-seq) data from the NCBI BioProject database (accession number: PRJNA806849) to conduct specific analyses targeting the *Mups* and *Ces1s* gene regions [32]. Our analysis revealed significant NRF2 binding peaks near both the *Mup7* and *Ces1g* gene regions (Figure 6H). Although these peaks were not directly localized to the gene promoter regions, subsequent analysis indicated the presence of H3K27ac modification signals in the adjacent areas (Figure S9) [32,33].

## 4. Discussion

Atherosclerosis is a major cause of various cardiovascular diseases and a contributor to acute cardiovascular events such as myocardial infarction and stroke [34]. Emerging evidence indicates that the NRF2 signaling pathway plays a regulatory role in the development of atherosclerosis [35,36,37]. In this study, we established hepatocyte-specific *Nrf2*-deficient mice in the *ApoE*-KO background, and the results provide evidence that NRF2 in hepatocytes can promote the pathological progression of atherosclerosis. Indeed, *Nrf2* deficiency reduces circulating levels of total cholesterol and LDL cholesterol and can alter lipid profiles. Furthermore, there were also profound regulatory effects on the transcriptome and proteome in terms of the number of genes affected and the magnitude of changes in gene expression, reflecting in part significant alterations in pathways related to cholesterol metabolism, lipid accumulation, and cholesterol transport.

There are conflicting views on the role of NRF2 in atherosclerosis based on *Nrf2*-knockout studies. Global *Nrf2*-knockout mice exhibited a significant 50% reduction in the degree of aortic atherosclerosis compared with littermate controls. NRF2 in multiple cells is involved in developing atherosclerosis, and systemic knockout mouse models fail to distinguish the functions performed by different cells. Therefore, cell-specific *Nrf2* deficiency models are widely used in studies of complicated disease investigation. Endothelial cell-specific *Nrf2* knockdown in the context of *ApoE*-KO exacerbates mouse atherosclerotic lesions by promoting oxidative damage and inflammatory responses in endothelial cells [21]. In aortic smooth muscle cells, it was reported that NRF2 adaptively activates in response to a wide range of stressors, including oxidized low-density lipoproteins and inflammatory cytokines. Lacking *Nrf2* can lead to impaired cellular resistance to oxidative stressors, contributing to the development of age-related vascular diseases, including atherosclerosis [38]. Specific *Nrf2*-knockout in bone marrow-derived cells increased the extent of atherosclerosis in *LDLR*-KO mice [23]. However, *Nrf2* expression in macrophages may paradoxically accelerate the progression of atherosclerosis by upregulating CD36 expression, thereby promoting lipid uptake and foam cell formation [14]. In summary, atherosclerosis was attenuated in mice with global *Nrf2*-knockout, whereas NRF2 in various cells could protect against atherogenesis by responding to antioxidant damage and inflammation. This suggests that there may be other cell types in which NRF2 plays an important function but has not been identified. In the current study, we found that atherosclerotic lesions were attenuated in hepatocyte *Nrf2* deficiency mice in the *ApoE*-KO background.

In the *ApoE*-KO mouse background, we found that in male mice, *Nrf2*(H)-KO mice had low plasma cholesterol levels compared with controls, and a significant difference between the two groups appeared around 24 weeks as well as in 60-week-old mice. Similar phenotypes were observed in female mice, even less pronounced than in the male mouse model. To explore the mechanism of the difference, we performed a metabolomic analysis of plasma in 24-week-old male mice and found differences in plasma lipid profiles due to the absence of hepatocyte-specific *Nrf2*. Plasma from mice with hepatocyte-specific *Nrf2* deletion had reduced glycerophospholipids and elevated free fatty acid lipids. Glycerophospholipids, important components of lipoproteins, are increased in *ApoE*-KO mice, whereas hepatocyte *Nrf2*-specific deletion results in lower plasma glycerophospholipids, which attenuates the development of atherosclerosis [39], and which are generated and transformed during the evolution of atherosclerosis and are closely related to the development of arterial inflammation, disease progression, and complications. Thus, a reduction in plasma levels of phospholipid metabolites is closely associated with a reduction in the degree of atherosclerosis. However, the role of the type and amount of fatty acid intake in atherosclerosis has been debated [40,41]. Notably, unsaturated fatty acids have been shown to reduce blood triglyceride levels and total blood cholesterol levels. The level of arachidonic acid, an important unsaturated fatty acid, was significantly reduced in *Nrf2*-LoxP;*ApoE*-KO, whereas it was not significantly altered in *Nrf2*(H)-KO;*ApoE*-KO mice, which may allow for a reduction in the degree of atherosclerosis in *Nrf2*(H)-KO;*ApoE*-KO mice, following the results we observed.

After pathological staining of the liver and quantifying cholesterol and triglyceride levels, we found that male *Nrf2*(H)-KO;*ApoE*-KO mice showed a moderate lipid accumulation in the liver with macrophage infiltration. We hypothesize that if *Nrf2* deficiency in hepatocytes reduces the efflux of cholesterol from the liver to the bloodstream, the degree of atherosclerosis in mice will be reduced, with concomitant hepatic lipid retention.

We performed transcriptome and proteome sequencing in mouse livers to elucidate the hepatocyte-specific mechanism of NRF2 in atherogenesis. The NRF2-affected pathways were mainly focused on cholesterol metabolism. Co-analysis of the sequencing results showed that the expression level of the carboxylesterase family was significantly reduced after *Nrf2* deficiency. Carboxylesterases (CESs) are primarily known to be involved in the detoxification and metabolism of drugs and environmental toxicants [42,43,44] as well as hydrolyzing endogenous esters and thioesters. It was demonstrated that human CES1 induces VLDL assembly and secretion in the liver in transgenic mice [45]. In addition, hepatocyte CES1 can be involved in cholesterol processing and influence the development of atherosclerosis [19]. *Ces1* is a continuous gene cluster with high gene sequence similarity. Several single *Ces1*-knockout mouse models (*Ces1c*, *Ces1d* and *Ces1g*) have been generated for drug and lipid metabolism studies [45,46]. It has been hypothesized that the endoplasmic reticulum luminal localization of LDL-associated *Ces1d* serves to mobilize lipids in order to provide substrates for VLDL assembly through the “hydrolysis/re-esterification cycle” process [47]. ChIP-seq confirmed that anti-NRF2 ChIP enriched a site in carboxylesterase 1G (*Ces1g*) [32]. As shown in Figure 6A, we therefore speculate that hepatocyte *Nrf2*-specific deletion affected the expression of the hepatic carboxylesterase family, which may influence the secretion of cholesterol from the liver into the bloodstream.

Major urinary proteins (MUPs) belong to a family of lipid transport proteins, and exhibited significantly higher expression levels in male mice. MUPs are mainly synthesized by the liver and secreted into the bloodstream, where they function as molecular chaperones for lipophilic compounds such as pheromones [48]. They have the structure of binding active lipophilic small molecules, and they are likely to affect glycolipid metabolism by binding lipophilic small molecules with glycolipid metabolism-regulating function [49,50]. In the present study, *Nrf2*(H)-KO mice showed a significant reduction in the expression of hepatic and plasma MUPs, consequently leading to a relative reduction in the transfer of lipids from the liver into the bloodstream, which may contribute to the accumulation of lipids in the liver as well as the attenuation of atherosclerosis. Sarah Greve et al. showed several upstream regulators involved in lipid metabolism and oxidative stress predicted by Ingenuity Pathway Analysis (IPA) in transcriptome sequencing of liver tissues from *Mup*-KO mice. The results indicated that NRF2 was the most relevant upstream liver regulator activated in male *Mup*-KO mice [51]. As members of lipid carrier proteins (LCNs), mouse MUPs are evolutionarily conserved, and they perform functions like human LCN9, LCN11, and LCN2 [49]. Previous studies have shown that recombinant MUP1 is functional even in primary human cell lines, suggesting that there may be a receptor for MUP1 or its signaling cascade in human hepatocytes, or that MUP1 shares a common receptor with LCNs [25].

The gene enhancer increases the promoter activity of target genes through specific transcription factors. Accumulated evidence reveals that the enhancers are marked by epigenetic modifications, such as mono-methylation at H3 lysine 4 (H3K4me1) and acetylation at H3 lysine 27 (H3K27ac) [52]. In the present study, ChIP-seq data analysis revealed that significant NRF2-binding peaks were detected in the vicinity of the *Mup7* and *Ces1g* gene loci, and H3K27ac modification signals were present in the regions adjacent to these binding peaks [32,33]. Collectively, these data provide hypothesis-generating evidence suggesting that NRF2 may potentially regulate the transcription of target genes, such as *Mup7* and *Ces1g*, through binding to their putative enhancer regions. This provides a theoretical foundation for future direct validations. Additionally, the lack of direct functional validation (e.g., ChIP-qPCR) for the ChIP-seq analyses represents the limitation.

In conclusion, hepatocyte *Nrf2* deficiency diminished plasma cholesterol levels, and consequently, influenced the initiation and development of atherosclerosis, in which the carboxylesterase enzymes and lipocalin family may be involved. The study highlights the need for future studies in human models using NRF2 regulators and inducible models to establish clinical relevance. The current study establishes the foundation for considering hepatic NRF2 as a target to control hypercholesterolemia and prevent the development of atherosclerosis.

## Data Availability

The original contributions presented in this study are included in the article/Supplementary Material. Further inquiries can be directed to the corresponding author. The data presented in this study are available in GEO, reference number [GSE346814]. The data were derived from the following resources available in the public domain: NCBI BioProject database: https://www.ncbi.nlm.nih.gov/bioproject/PRJNA806849/ (accessed on 2 July 2026); https://www.ncbi.nlm.nih.gov/bioproject/PRJNA345270/ (accessed on 2 July 2026).

## Supplementary Materials

The following supporting information can be downloaded at: https://www.mdpi.com/article/10.3390/antiox15091183/s1, Table S1. Primer sequences for quantitative RT-PCR. Figure S1. Plasma lipid profiles in male mice. Figure S2. Plasma lipid profiles in 24-week-old female mice. Figure S3. Plasma lipid profiles in 60-week-old male mice. Figure S4. RNA-seq analysis of liver tissues. Figure S5. Proteomics analysis of liver tissues. Figure S6. Proteomics analyses highlight significant changes following *Nrf2* hepatocyte-specific knockdown in the context of *ApoE*-KO. Figure S7. GSEA analysis. Figure S8. Immunohistochemical staining and RT-qPCR. Figure S9. ChIP-seq visualization analysis. Figure S10. Hepatic VLDL secretion and LDL uptake. Figure S11. Oil-red O staining of the aorta. Figure S12. Schematic diagram of the experimental design.
