# Supplementary material for: Hepatocyte-Specific Nrf2 Deficiency Alters Cholesterol Metabolism, Leading to Mitigated Atherosclerosis in ApoE-Knockout Mice
Source: Antioxidants (Basel). 2026 Sep 17;15(9):1183. doi: 10.3390/antiox15091183 (PMC13603266; doi:10.3390/antiox15091183)
Supplement: Supplementary file 1 [file antioxidants-15-01183-s001.zip › antioxidants-4446221-supplementary.pdf]

Hepatocyte-specific *Nrf2* deficiency alters cholesterol metabolism leading to mitigated atherosclerosis in *ApoE*-knockout mice

Junying Jiao[1,2,3], Ning Xu[3], Zhixuan Hong[1,2,3], Lei Chang[4], Derong Huang[1,2,3], Yang Yu[1,2,3], Zhendi Wang[3], Yibai Li[3], Jiaxin Yu[3], Juntao Guo[3], You Wang[3], Lirun Kuang[5], Yong Wang[5], Bei Yang[6], Rui Zhao[7], Yongyong Hou[1,2,3], Huihui Wang[1,2,3], Qiang Zhang[8], Ping Xu[4], Yuanyuan Xu[1,2,3], Jingbo Pi[1,2,3,*], Jingqi Fu[1,2,3,*]

Table S1. Primer sequences for quantitative RT-PCR

Gene	Accession number	Forward	Reverse
β-actin	NM_007393.5	5 ' - ACCCTGAAGTACCCATCGAG - 3 '	5 ' - ACATGATCTGGGTCATCTTCTCG - 3 '
Nrf2	NM_010902.5	5 ' - GGCAGTGTCCTCCTCATTCC- 3 '	5 ' - TCCGGTCCTTTGGCTTTCT- 3 '
Mups	NM_001347129.1	5 ' - GAAGCTAGTTCTACGGGAAGGA- 3 '	5 ' - AGGCCAGGATAATAGTATGCCA- 3 '
Gstm1	NM_001374678.1	5 ' -ATACTGGGATACTGGAACGTCC- 3 '	5 ' -AGTCAGGGTTGTAACAGAGCAT- 3 '
Ces1b	NM_001081372.1	5 ' -TACCTCCCCTGTTTTCCGAAG- 3 '	5 ' -GATGCTCCGCCTGTCATCAAT- 3 '
Ces1d	NM_053200.2	5 ' -ACAACAGATGTAAAGCCCATTGC- 3 '	5 ' -GCCTTTGGCAGAACTACTCCAT- 3 '
Ces1f	NM_144930.2	5 ' -TGTGAGGCCAATTACTGAGCA- 3 '	5 ' -CGAACTGGTCGCTATTTTTGGTA- 3 '
Ces1g	NM_021456.4	5 ' -CTGCTTGCTTGAGTCTGGGAC- 3 '	5 ' -TTGGTAGCACAAAGGAGGGTA- 3 '
Abcg5	NM_031884.2	5 ' - TGGATCCAACACCTCTATGCTAAA - 3 '	5 ' -GGCAGGTTTTCTCGATGAACTG - 3 '
Abcg8	NM_001286005.1	5 ' - TGCCCACCTTCCACATGTC - 3 '	5 ' - ATGAAGCCGGCAGTAAGGTAGA - 3 '

Figure S1

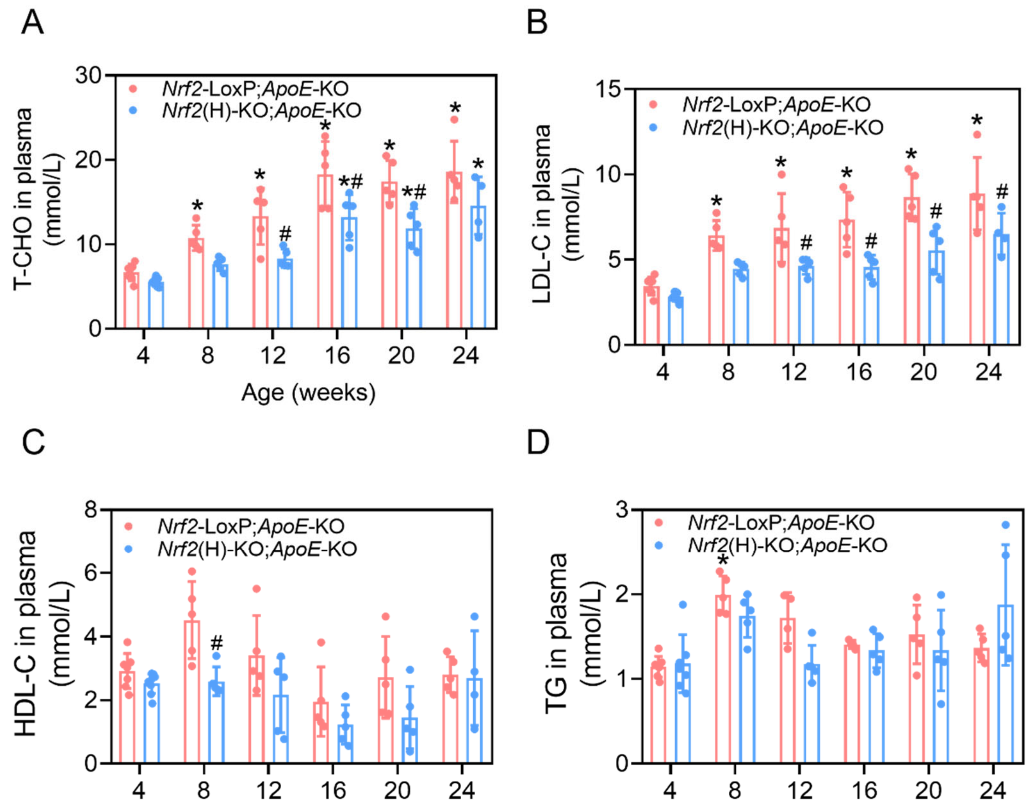

Figure. S1. Plasma lipid profiles in male mice. The plasma was harvested from tail vein at *ad libitum* conditions. Plasma total cholesterol (T-CHO, A), low-density lipoprotein cholesterol (LDL-C, B), high-density lipoprotein cholesterol (HDL-C, C) and total triglyceride (TG, D) levels were monitored in male mice regularly. n = 4-6, $^{*}p < 0.05$ vs the same group mice at 4-week-old, $^{\#}p < 0.05$ vs the same age of *Nrf2*-LoxP;*ApoE*-KO mice.

Figure S2

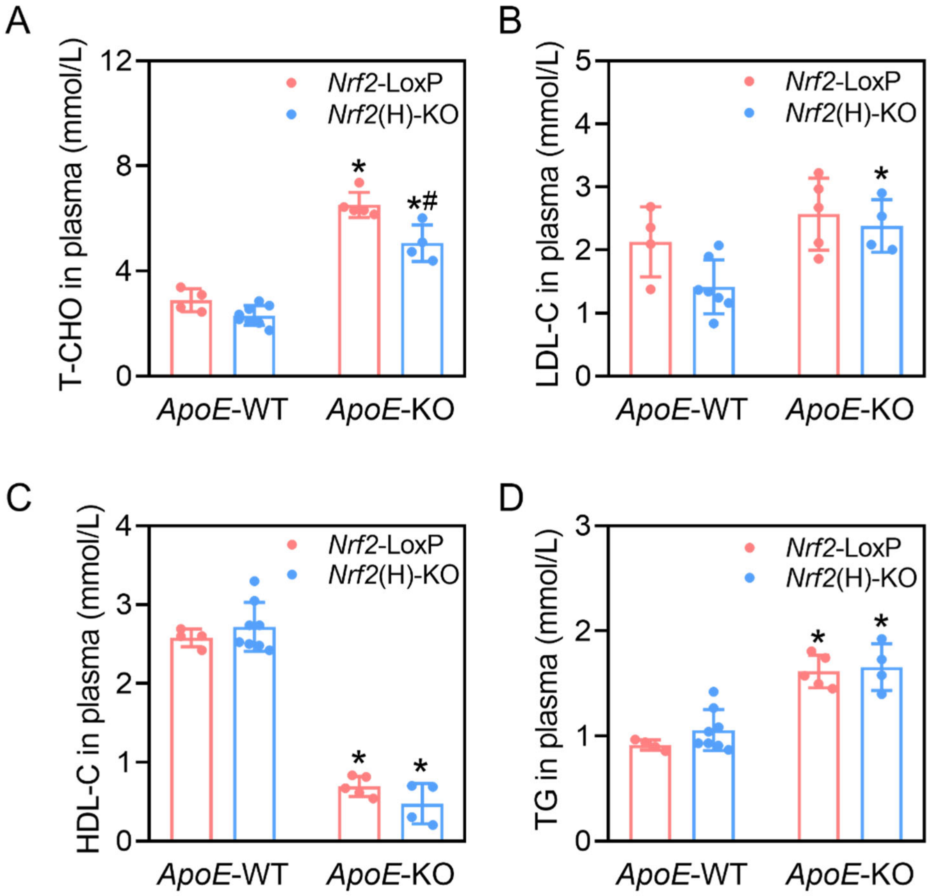

Figure. S2. Plasma lipid profiles in 24-week-old female mice. Plasma samples were harvested when the mice were sacrificed under fasting conditions. Total cholesterol (T-CHO, A), low-density lipoprotein cholesterol (LDL-C, B), high-density lipoprotein cholesterol (HDL-C, C), total triglyceride (TG, D) of plasma in 24-weeks female mice. n = 4-8, $^{*}p < 0.05$ vs the same genotype of *ApoE*-WT background , $^{\#}p < 0.05$ vs *Nrf2*-LoxP; *ApoE*-KO mice.

Figure S3

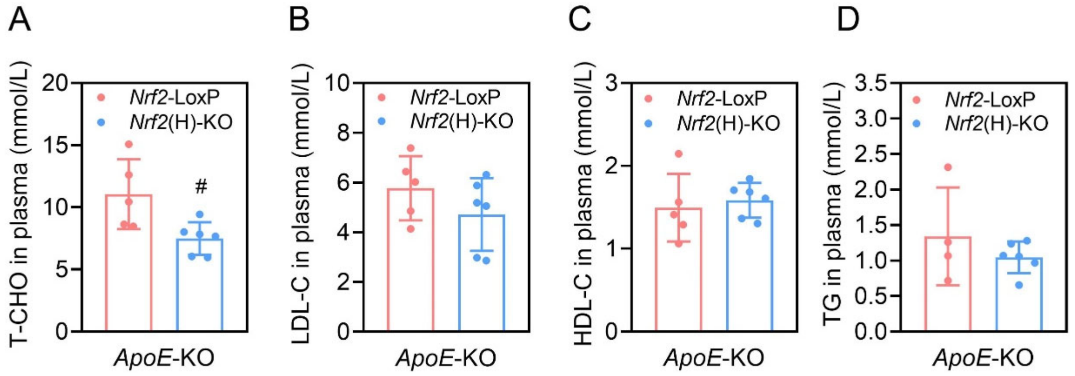

Figure. S3. Plasma lipid profiles in 60-week-old male mice. Plasma samples were harvested when the mice were sacrificed under fasting conditions. Total cholesterol (T-CHO, A), low-density lipoprotein cholesterol (LDL-C, B), high-density lipoprotein cholesterol (HDL-C, C), total triglyceride (TG, D) of plasma in 60-weeks male mice. n = 4-6, $^{\#}p < 0.05$ vs *Nrf2*-LoxP; *ApoE*-KO mice.

Figure S4

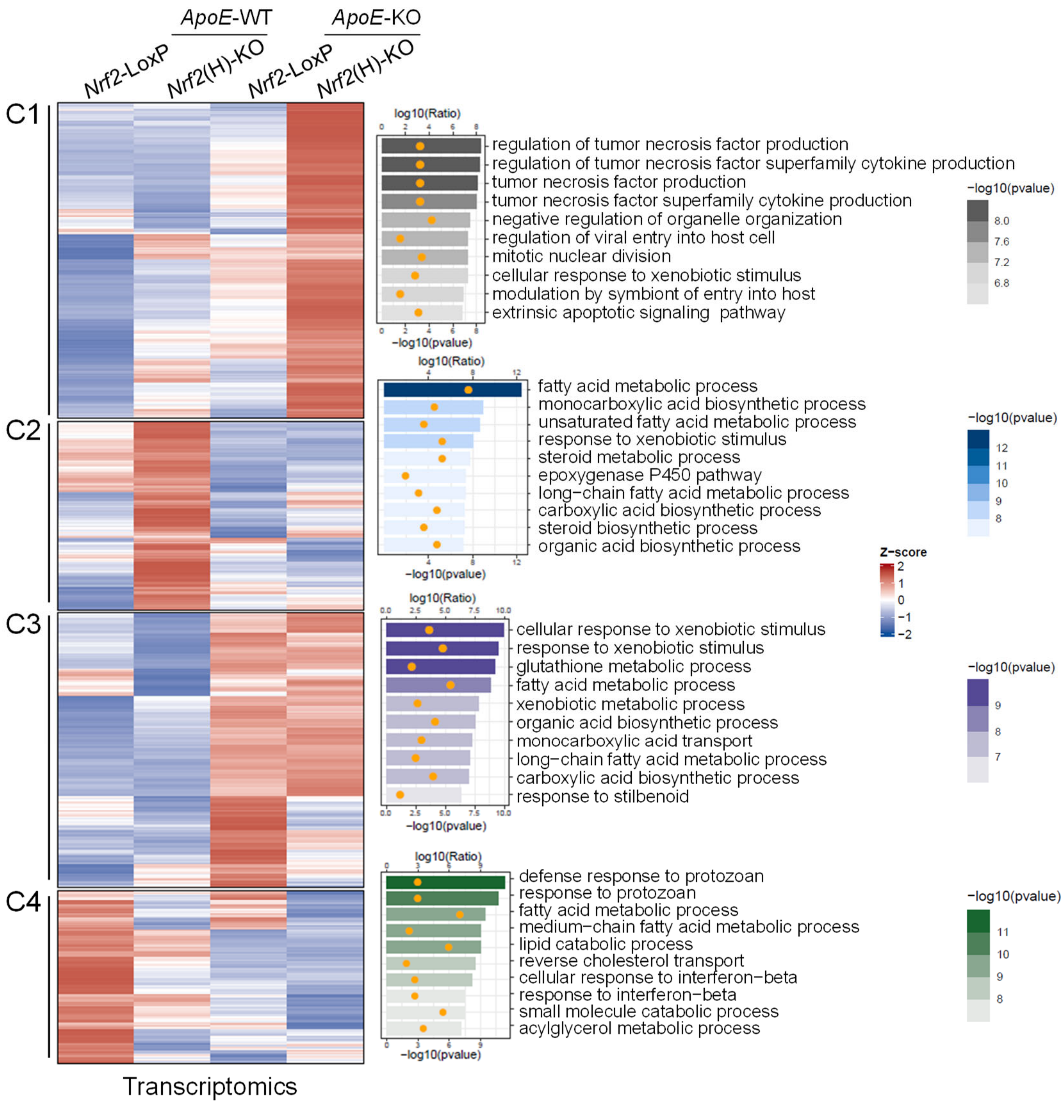

Figure. S4. RNA-seq analysis of liver tissues. The results of a fuzzy c-means clustering analysis combined with a gene ontology enrichment analysis of DEGs are shown. Fuzzy c-means clustering classified all DEGs into four clusters.

C1: regulation of tumor necrosis factor production; regulation of tumor necrosis factor; superfamily cytokine production; tumor necrosis factor production; tumor necrosis factor superfamily cytokine production; negative regulation of organelle organization; regulation of viral entry into host cell; mitotic nuclear division; cellular response to xenobiotic stimulus; modulation by symbiont of entry into host; extrinsic apoptotic signaling pathway.

C2: fatty acid metabolic process; monocarboxylic acid biosynthetic process; unsaturated fatty acid metabolic process; response to xenobiotic stimulus; steroid metabolic process; epoxygenase P450 pathway; long−chain fatty acid metabolic process; carboxylic acid biosynthetic process; steroid biosynthetic process; organic acid biosynthetic process.

C3: cellular response to xenobiotic stimulus; response to xenobiotic stimulus; glutathione metabolic process; fatty acid metabolic process; xenobiotic metabolic process; organic acid biosynthetic process; monocarboxylic acid transport; long−chain fatty acid metabolic process; carboxylic acid biosynthetic process; response to stilbenoid.

C4: defense response to protozoan; response to protozoan; fatty acid metabolic process; medium−chain fatty acid metabolic process; lipid catabolic process; reverse cholesterol transport; cellular response to interferon−beta; response to interferon−beta; small molecule catabolic process; acylglycerol metabolic process.

Figure S5

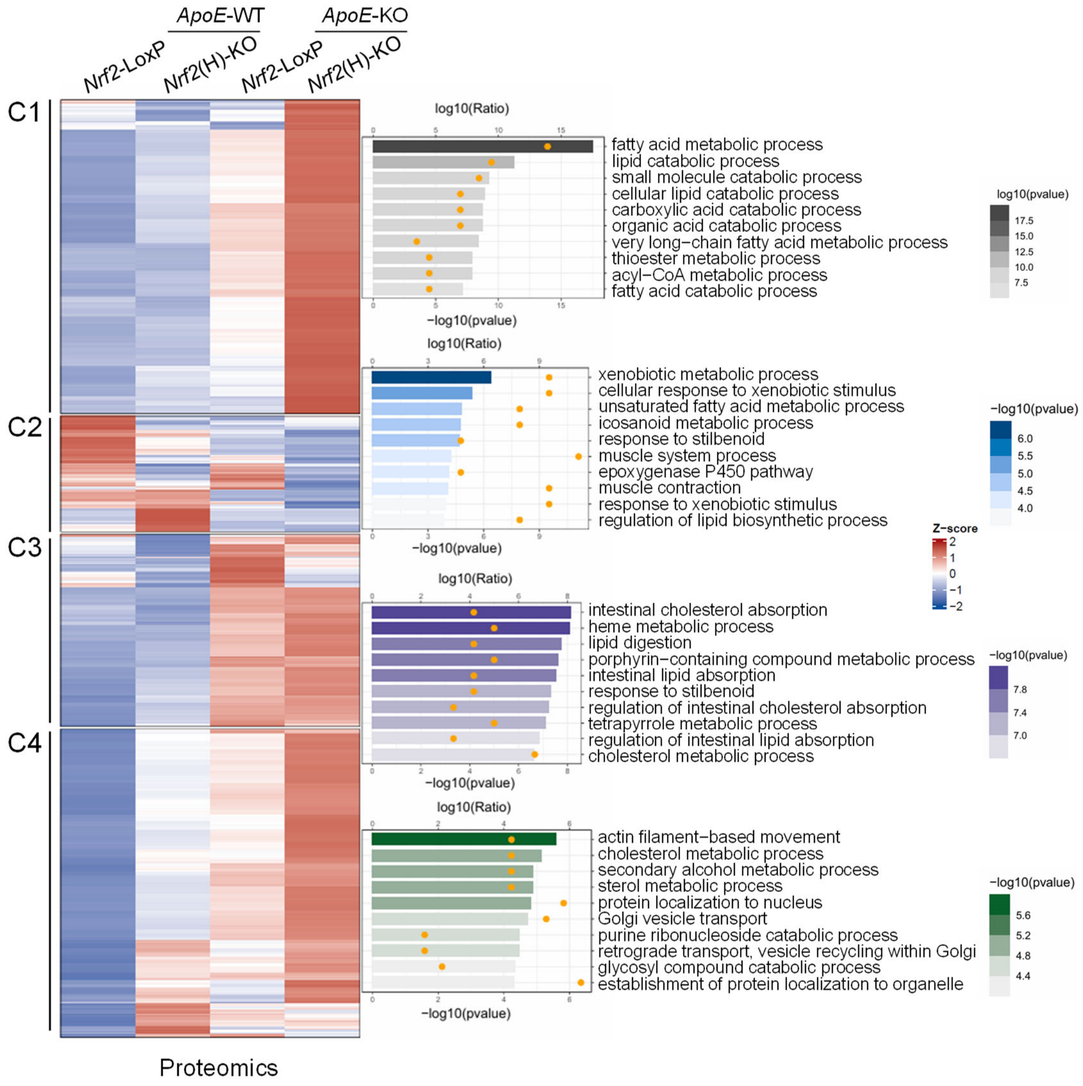

Figure. S5. Proteomics analysis of liver tissues. The results of a fuzzy c-means clustering analysis combined with a protein ontology enrichment analysis of DEPs are shown. Fuzzy c-means clustering classified all DEPs into four clusters.

C1: fatty acid metabolic process; lipid catabolic process; small molecule catabolic process; cellular lipid catabolic process; carboxylic acid catabolic process; organic acid catabolic process; very long−chain fatty acid metabolic process; thioester metabolic process; acyl−CoA metabolic process; fatty acid catabolic process.

C2: xenobiotic metabolic process; cellular response to xenobiotic stimulus; unsaturated fatty acid metabolic process; icosanoid metabolic process; response to stilbenoid; muscle system process; epoxygenase P450 pathway; muscle contraction; response to xenobiotic stimulus; regulation of lipid biosynthetic process.

C3: intestinal cholesterol absorption; heme metabolic process; lipid digestion;

porphyrin−containing compound metabolic process; intestinal lipid absorption; response to stilbenoid; regulation of intestinal cholesterol absorption; tetrapyrrole metabolic process; regulation of intestinal lipid absorption; cholesterol metabolic process.

C4: actin filament−based movement; cholesterol metabolic process; secondary alcohol metabolic process; sterol metabolic process; protein localization to nucleus; Golgi vesicle transport; purine ribonucleoside catabolic process; retrograde transport, vesicle recycling within Golgi; glycosyl compound catabolic process; establishment of protein localization to organelle.

Figure S6

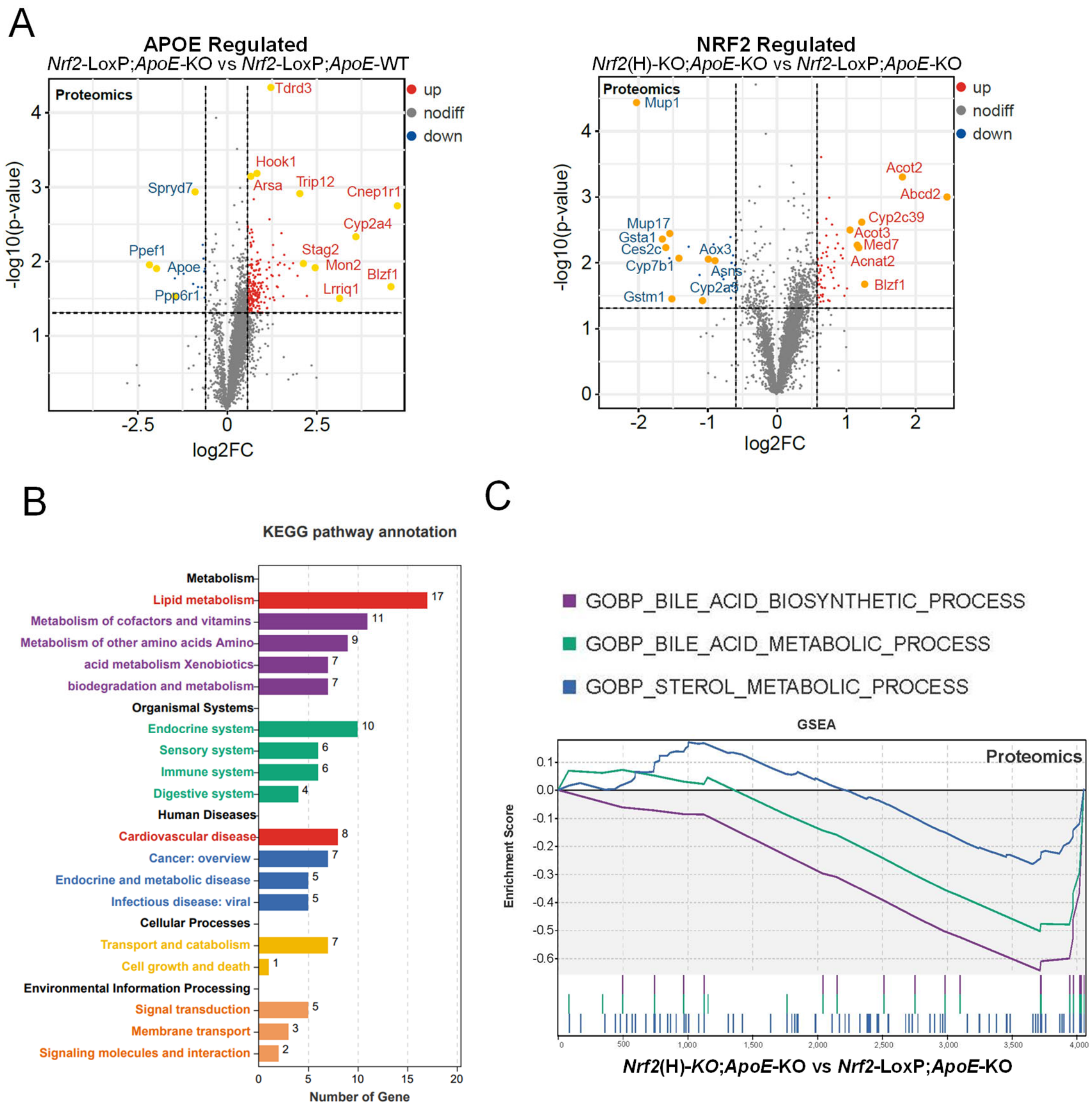

Figure. S6. Proteomics analyses highlight significant changes following *Nrf2* hepatocyte-specific knockdown in the context of *ApoE*-KO. (A) Proteins regulated by APOE (*Nrf2*-LoxP;*ApoE*-KO vs *Nrf2*-LoxP;*ApoE*-WT) and NRF2 (*Nrf2*(H)-KO; *ApoE*-KO vs *Nrf2*-LoxP; *ApoE*-KO) respectively in proteomics. **(B)** KEGG enrichment analysis of NRF2-regulated proteins. **(C)** GSEA shows that NRF2 downregulates Bile Acid Process and Sterol metabolic network.

Figure S7

A

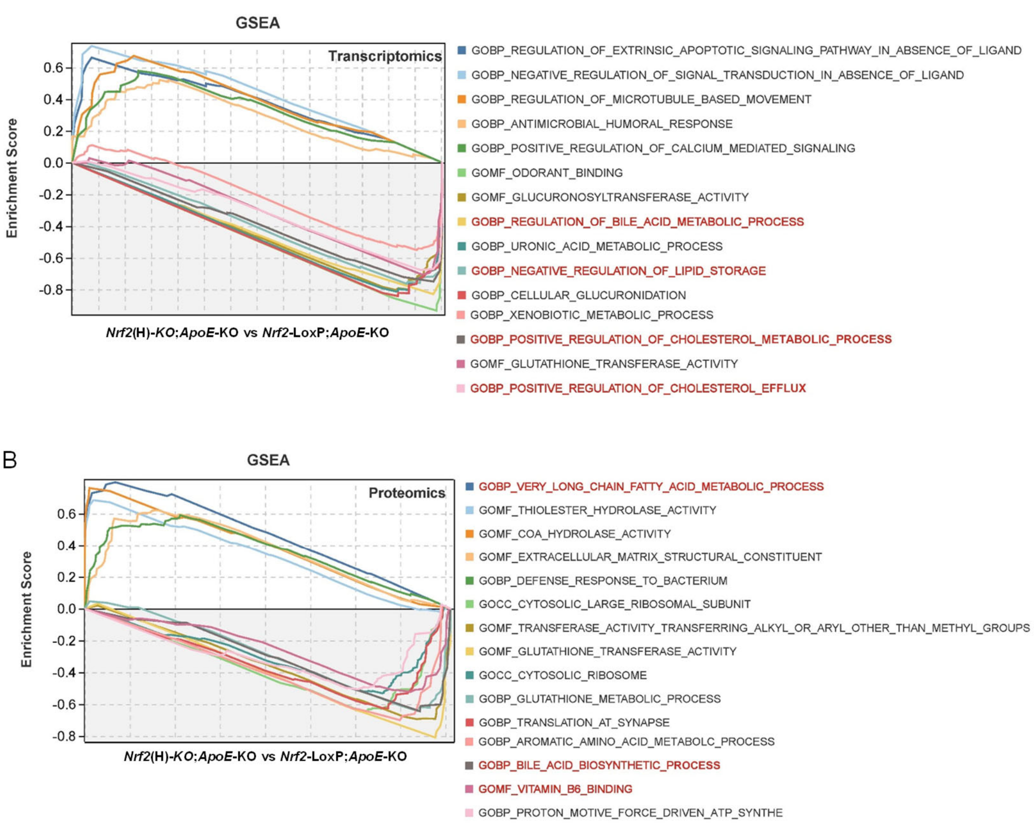

Figure. S7. GSEA analysis. (A) GSEA analysis of NRF2-regulated (*Nrf2*(H)-KO; *ApoE*-KO vs *Nrf2*-LoxP; *ApoE*-KO) genes, and it shows the top 15 pathways. **(B)** GSEA analysis of NRF2-regulated (*Nrf2*(H)-KO; *ApoE*-KO vs *Nrf2*-LoxP; *ApoE*-KO) proteins, and it shows the top 15 pathways.

Figure S8

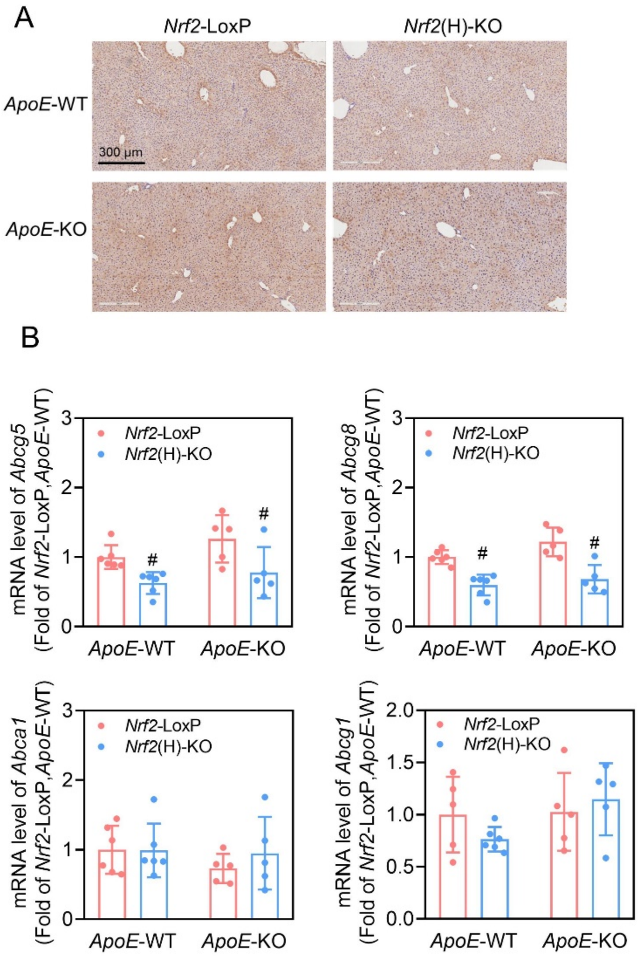

Figure. S8. Immunohistochemical staining and RT-qPCR (A) Immunohistochemical staining of mouse liver for CES1. (B) Expression levels of genes related to bile acid metabolism. n = 5-6, $^{\#}p < 0.05$ vs *Nrf2*-LoxP group of the same *ApoE* genotype.

Figure S9

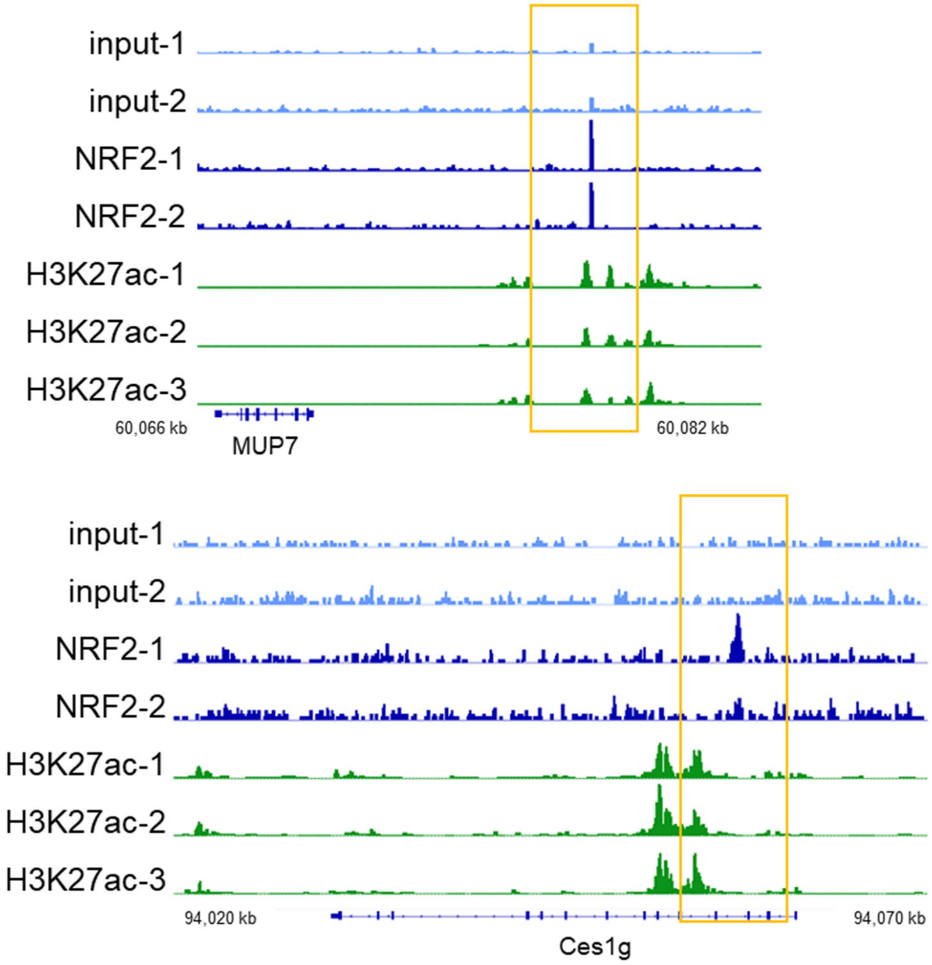

Figure. S9. ChIP-seq visualization analysis. IGV tracks for *Mup7 and Ces1g* from ChIP-seq analysis. The yellow boxes indicated significant binding peaks. (NRF2 ChIP-seq: PRJNA806849 and H3K27ac ChIP-seq: PRJNA345270).

Figure S10

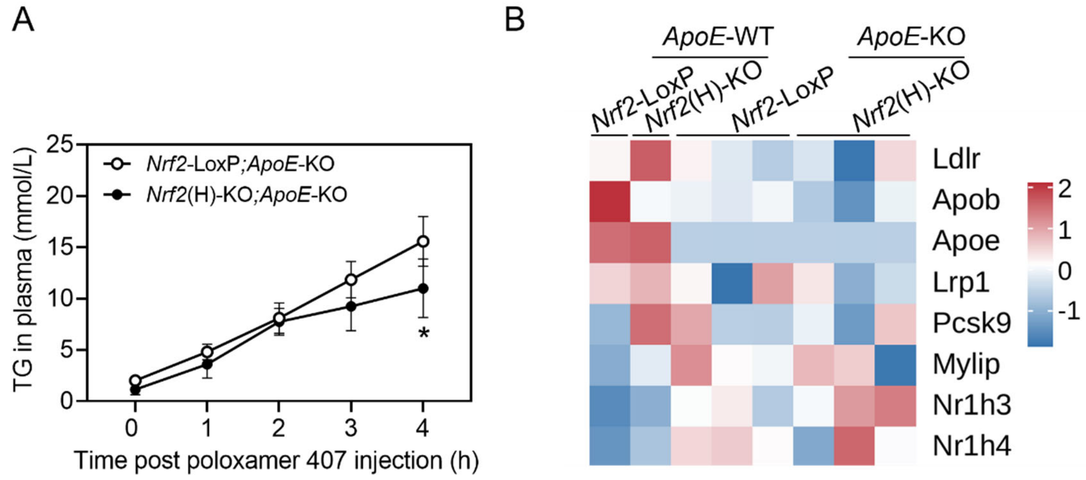

Figure. S10 Hepatic VLDL secretion and LDL uptake. (A) Plasma TG levels were determined by the Poloxamer-407 injection test. (B) Heatmap of LDL uptake-related genes (*Ldlr, Apob, Apoe, Lrp1, Pcsk9, Mylip, Nr1h3, Nr1h4*). n = 3, $^{*}p < 0.05$ vs the same genotype of *ApoE*-WT background.

Figure S11

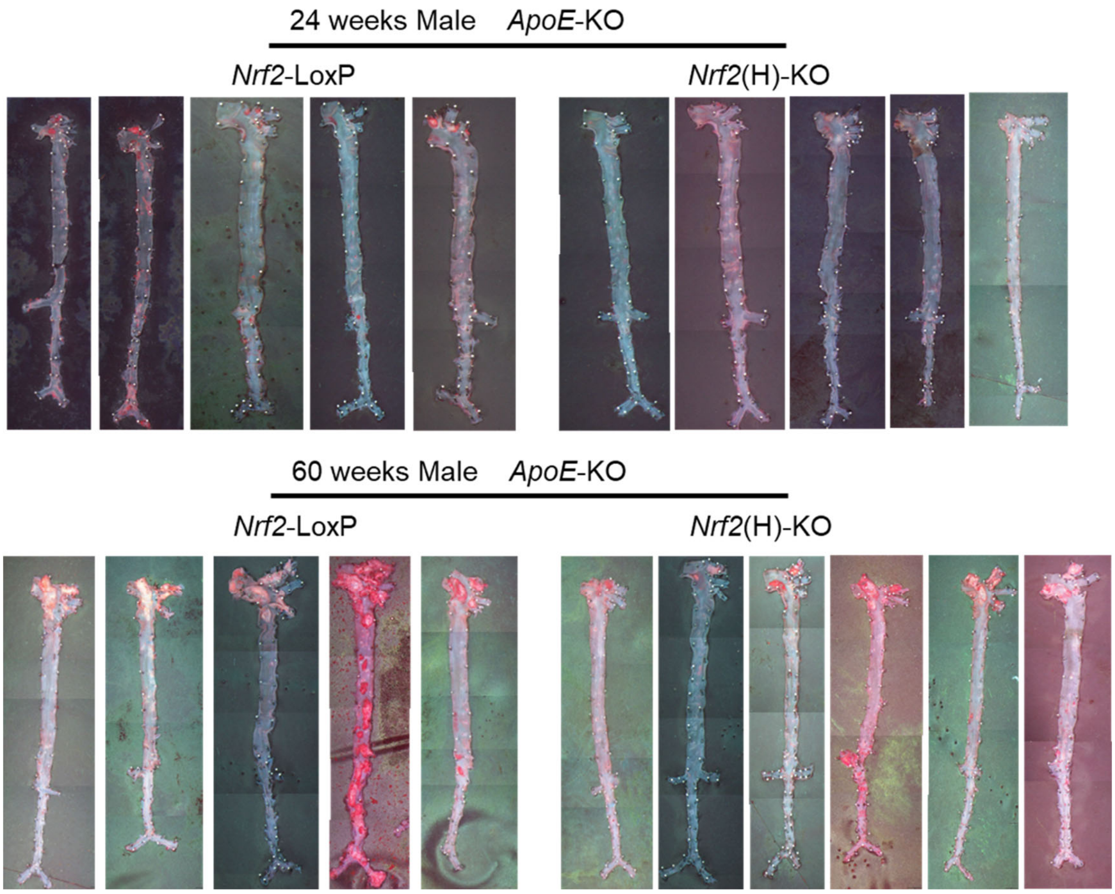

Figure. S11 Oil Red O staining of the aorta. Oil Red O staining of the aorta in mice of different ages (24 weeks and 60 weeks) of males. n = 5-6.

Figure S12

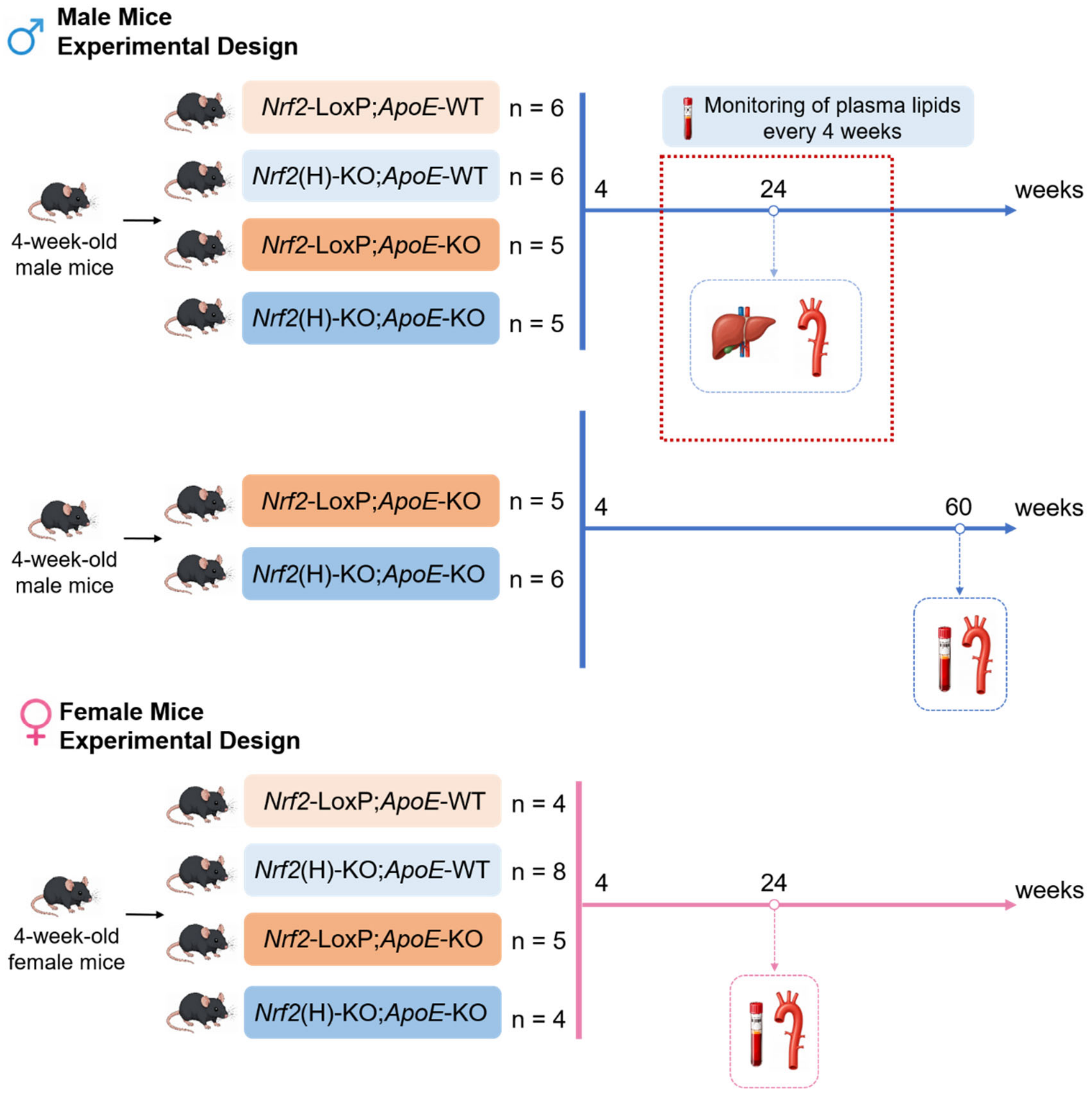

Figure. S12 Schematic diagram of the experimental design. In this study, 24-week-old male mice, 60-week-old male mice and 24-week-old female mice were utilized. For the experimental design, mice were randomly divided into four groups: (1) *Nrf2*-LoxP;*ApoE*-WT; (2) *Nrf2*(H)-KO;*ApoE*-WT; (3) *Nrf2*-LoxP;*ApoE*-KO; and (4) *Nrf2*(H)-KO;*ApoE*-KO. Livers and aortas were harvested at the respective time points.
